# Intra‐annual transfer of hydrogen and oxygen isotopic signals from water and sugar precursors to tree rings: Processes and mechanisms

**DOI:** 10.1111/nph.71260

**Published:** 2026-05-13

**Authors:** Haoyu Diao, Meisha Holloway‐Phillips, Xin Song, Fabian Bernhard, Peter Waldner, Kerstin Treydte, Matthias Saurer, Georg von Arx, Arthur Gessler, Katrin Meusburger, Marco M. Lehmann

**Affiliations:** ^1^ Swiss Federal Institute for Forest, Snow and Landscape Research WSL Birmensdorf 8903 Switzerland; ^2^ College of Life Sciences and Oceanography Shenzhen University Shenzhen 518060 China; ^3^ Oeschger Centre for Climate Change Research University of Bern Bern 3012 Switzerland; ^4^ Institute of Geography University of Bern Bern 3012 Switzerland; ^5^ Department of Environmental Systems Science ETH Zürich Zürich 8092 Switzerland

**Keywords:** broadleaf, cellulose, conifer, hydrogen isotopes, isotopic exchange, nonstructural carbohydrate, oxygen isotopes, tree rings

## Abstract

- The oxygen (δ^18^O) and hydrogen (δ^2^H) isotope compositions of leaf and xylem water shape tree‐ring isotope baselines, while the fraction of sugars undergoing isotopic modification downstream of leaves (*f*_O_, *f*_H_) determines the dominant hydrologic signal. However, limited information on the seasonal dynamics of these isotope sources and on the drivers of *f* variation constrains tree‐ring isotope interpretation.
- We measured intra‐annual δ^18^O and δ^2^H in stem water, sugar, starch, and tree‐ring α‐cellulose of beech and spruce over two growing seasons. Using modelled leaf water δ^18^O and δ^2^H, we estimated seasonal *f* values and examined their relationships with nonstructural carbohydrate concentrations and climate.
- Tree rings primarily recorded δ^18^O and δ^2^H signatures of leaf water, despite seasonal changes in *f*_O_ and *f*_H_. We found no clear transfer of intra‐annual xylem water isotopic signals into sugars, starch, or cellulose. Seasonal *f*_O_ and *f*_H_ can be negatively correlated. Both were related to climate variables, but only *f*_O_ was correlated with nonstructural carbohydrate concentrations.
- Thus, isotopic fractionation downstream of leaves does not always override the seasonal imprint of leaf water in tree rings. These findings provide insight into the controls on the *f*_O_–*f*_H_ covariation, supporting more robust interpretations of climate variability from tree‐ring isotope records.

## Graphical abstract

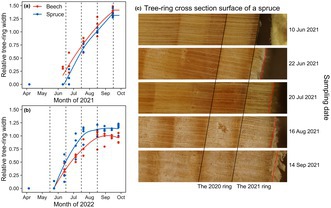

## Introduction

The stable isotope compositions of oxygen (δ^18^O) and hydrogen (δ^2^H) in tree‐ring cellulose have become powerful tools for reconstructing climate (Treydte *et al*., 2024), assessing forest water dynamics (Diao *et al*., 2025), and evaluating plant performance (Vitali *et al*., 2024). This is because climatic variables influence isotopic variation in xylem and leaf water (Cernusak *et al*., 2016), which are major sources of O and carbon‐bound H in plant compounds, including cellulose. Consequently, there is strong interest in understanding the isotope processes occurring during tree‐ring formation and the relative contribution of different isotopic signals within plant tissues (Lin *et al*., 2022; Gessler *et al*., 2024; Wieloch *et al*., 2025).

It has long been recognised that the leaf water isotopic signal initially imprinted on photosynthetic sugars can be altered during downstream metabolism in ‘sink’ tissues (Yakir & DeNiro, 1990; Luo & Sternberg, 1992). The isotope composition of tree‐ring cellulose (δ_C_) is therefore determined by (1) the isotope composition of leaf and xylem water, (2) the fraction of sugar substrates that undergo isotopic modification after export, and (3) isotopic fractionation resulting from kinetic and equilibrium isotope effects during spontaneous or enzyme‐mediated reactions, each varying both within and between years (Gessler *et al*., 2009; Offermann *et al*., 2011). These drivers are represented in a two‐pool cellulose isotope mixing model (Yakir & DeNiro, 1990; Barbour & Farquhar, 2000; Roden *et al*., 2000). As the fraction of sugars that undergo post‐export isotopic modification (*f*) cannot be directly measured, it is often inferred by inverting this model using measured δ_C_ values and assumed isotope fractionation parameters (Holloway‐Phillips *et al*., 2022; Szejner *et al*., 2025). Even so, such attempts remain uncommon, leaving key gaps in understanding the relative influence of xylem and leaf water isotopic signals on tree‐ring cellulose.

Although the number of subannual studies measuring δ_C_ of different segments within a single tree‐ring is increasing (Treydte *et al*., 2014; Schubert & Jahren, 2015; Xu *et al*., 2016; Belmecheri *et al*., 2018; Szejner *et al*., 2020, 2025; Martínez‐Sancho *et al*., 2023), they remain relatively scarce compared to interannual studies, particularly for hydrogen isotopes (Nabeshima *et al*., 2018; Kagawa & Battipaglia, 2022). Yet, subannual studies are important because environmental and physiological processes vary seasonally during wood formation, and seasonally integrated signals may obscure key information (Schönbeck & Santiago, 2022). Studies have reported that intra‐annual δ^18^O_C_ signals originate from source water δ^18^O (including precipitation, soil water, and xylem water; Treydte *et al*., 2014; Schubert & Jahren, 2015; Martínez‐Sancho *et al*., 2023) and relative humidity (RH) (Szejner *et al*., 2020). The relative seasonal variability of these factors determines which signal dominates δ^18^O_C_ (Szejner *et al*., 2025; Kersti *et al*., 2026). For δ^2^H_C_, it has been reported in *Quercus crispula* that its intra‐annual pattern differs from that of δ^18^O_C_, with δ^2^H_C_ reaching its maximum at the beginning of each ring (Nabeshima *et al*., 2018). Although δ^18^O_C_ in cellulose generally retains hydrological signals better than δ^2^H_C_ at the interannual scale (Diao *et al*., 2025), it remains unclear whether this holds at the intra‐annual scale and whether the dominant hydrologic signal (leaf vs xylem water) is the same.

Intra‐annual variations in the fractional contribution of leaf and downstream isotopic signals in cellulose for oxygen and hydrogen (*f*_O_ and *f*_H_) have received less attention, especially for *f*_H_, leaving the reason for the often observed decoupling between δ^18^O and δ^2^H in tree‐ring (Vitali *et al*., 2022; Holloway‐Phillips *et al*., 2023; Diao *et al*., 2025) largely unexplored. For oxygen, post‐export isotopic modification occurs mainly through spontaneous or enzyme‐mediated equilibrium exchange between sugars and local water during hexose‐ and triose‐phosphate cycling (DeNiro & Cooper, 1989; Farquhar *et al*., 1998) (Supporting Information Fig. S1). The opportunity for oxygen isotopic exchange increases with sugar residence time in sink tissues (Hill *et al*., 1995; Barbour & Farquhar, 2000; Song *et al*., 2014). Hydrogen, however, follows different rules: although the key metabolic reactions were suggested to be the same as oxygen (Yakir & DeNiro, 1990; Augusti *et al*., 2006), hydrogen isotopic exchange cannot occur spontaneously and isotopic equilibrium is not always achieved due to intramolecular isotope transfer (Rose & O'Connell, 1961). In addition, many enzymes, particularly those involved in unidirectional reactions, associate with large normal kinetic isotope effects (KIEs) that do not involve water (Fig. S1). Thus, *f* is more accurately defined as the extent of ‘isotopic modification’ rather than only ‘isotopic exchange’. Recent studies further indicate that substantial isotopic modification can occur during phloem loading and transport, involving multiple tissues and metabolic pathways. Empirical evidence includes observations that phloem sugars sampled from petioles (Pan *et al*., 2024), twigs or branches (Gessler *et al*., 2013; Treydte *et al*., 2014; Fiorella *et al*., 2022), and stems (Offermann *et al*., 2011) are more depleted in ^18^O than leaf sugars. The differing mechanisms governing oxygen and hydrogen isotopic modifications that occur during carbon metabolism imply that positive covariation between *f*_O_ and *f*_H_ is not necessarily expected; therefore, oxygen and hydrogen isotope records in tree rings may capture different hydroclimatic signals. Indeed, studies evaluating both elements show inconsistent relationships between *f*_O_ and *f*_H_ – for example, positive covariation in the cellulose of seedlings but none across species comparisons of leaf tissue (Luo & Sternberg, 1992; Holloway‐Phillips *et al*., 2022).

Finally, growing evidence suggests that tree‐ring δ^18^O and δ^2^H values are also influenced by growth patterns, cambial age, and xylogenesis, all linked to seasonal carbon dynamics (Nabeshima *et al*., 2018; Szejner *et al*., 2020; Kagawa & Battipaglia, 2022; Martínez‐Sancho *et al*., 2023; Lehmann *et al*., 2024b). Given the distinct metabolic processes influencing *f*_O_ and *f*_H_, it is important to determine whether more commonly measured indicators of carbon use, such as nonstructural carbohydrate (NSC) concentrations, serve as useful proxies for post‐export isotopic modification. To date, only one study has linked *f*_H_ to the proportion of sugar relative to total NSC in leaves (Holloway‐Phillips *et al*., 2022).

To address these gaps, we investigated intra‐annual δ^18^O and δ^2^H values of water, sugar, starch, and α‐cellulose in stem tissue, together with stem‐xylem NSC content, in beech (*Fagus sylvatica* L.) and spruce (*Picea abies* (L.) H. Karst.) during two climatically contrasting growing seasons (2021: cool and wet; 2022: warm and dry). We first assessed the transfer of isotopic signals from water, sugars, and starch to cellulose at subannual resolution. We then applied the cellulose isotope model to explore how *f*_O_ and *f*_H_ relate to climate and NSC dynamics. Based on known biochemical differences between oxygen and hydrogen fractionation, we hypothesised that (1) isotopic signals from water sources and turnover of organic precursors are more strongly imprinted on oxygen than hydrogen in cellulose; (2) *f*_O_ and *f*_H_ are not necessarily positively correlated within a growing season; and (3) their relationships with climate and NSC differ between elements.

## Materials and Methods

### Study site

The study was conducted in 2021 and 2022 at a Long‐Term Forest Ecosystem Research (LWF) site in Bettlachstock, Switzerland (47.23° N, 7.42° E; 1130 m asl). The site is located on a steep slope (average gradient 66%) with soils classified as Rendzic Leptosols and/or Calcaric Cambisols (Walthert *et al*., 2003; Waldner *et al*., 2023). The soil water‐holding capacity from the surface to 100 cm depth was estimated as 138 l m^−2^; soil matrix potential measured at 15 cm depth was −540, −43, and −4 hPa at the 5th, 50th, and 95th percentiles, respectively, based on data averaged over 1998–2011 (von Arx *et al*., 2013). The dominant tree species are *Fagus sylvatica* L., *Picea abies* (L.) H. Karst., and *Abies alba* Mill. Tree density (diameter at breast height, DBH ≥ 12 cm) was 493.75 trees per hectare in 2011.

Gridded climate data (250‐m resolution) were obtained from Meteotest AG (Bern, Switzerland) and correspond closely with on‐site measurements (Didion *et al*., 2023). Long‐term (1981–2022) mean annual air temperature (*T*_air_) is 7.2°C, mean annual RH is 76%, and mean annual precipitation is 1364 mm. In 2021 and 2022, mean *T*_air_ was 7.2°C and 9.2°C, mean RH was 75% and 70%, and total precipitation was 1285 and 1085 mm, respectively.

### Stem core sampling, xylem water extraction, and stable isotope analysis

Four mature and healthy trees of *F. sylvatica* (beech) and four *P. abies* (spruce) were selected at the study site. The average DBH of sampled trees was 82 ± 8 cm for beech and 51 ± 6 cm for spruce. Stem cores (5 cm length, 5 mm diameter) were collected from those trees seven times in 2021 (April 6, June 10, June 22, July 20, August 16, September 14, and September 15) and nine times in 2022 (April 5, May 24, June 13, July 5, July 20, August 16, August 30, September 13, and September 26). Stem phloem and xylem tissues were separated in the field and both sealed in 12‐ml airtight glass vials (Labco Limited, Lampeter, UK).

Xylem water was extracted cryogenically at 80°C under < 0.05 mbar vacuum for 2 h (Diao *et al*., 2022; Bernhard *et al*., 2024). δ^18^O and δ^2^H values (δ^18^O_XW_ and δ^2^H_XW_) were measured using a cavity ring‐down spectrometer (L2140‐i) coupled to a microcombustion module (A0214; Picarro Inc., Santa Clara, CA, USA), with analytical precision of 0.5‰ for δ^18^O and 1.5‰ for δ^2^H.

### Determination of tree radial growth curves

After water extraction, surfaces perpendicular to the wood fibres were prepared using a lab microtome (Gärtner *et al*., 2015). High‐resolution images (6700 dots per inch (dpi)) of core surfaces were obtained with the Skippy system at WSL, equipped with a 61 MP camera (Sony Alpha 7R IV, Sony FE 90 mm f/2.8 Macro; lens, Tokyo, Japan) (Gärtner *et al*., 2024). Ring boundaries were clearly visible. For each core, the width of the current‐year ring (2021 or 2022) and the preceding ring were measured, and their ratio was used to quantify relative current‐year radial growth during each sampling date.

### Xylem sugar and starch concentration analysis

For each stem core, 2 cm of outer xylem tissue was oven‐dried and milled to fine powder using a ball mill (MM400, Retsch GmbH, Haan, Germany). Sugar and starch concentrations were determined following Wong (1990) as modified by Hoch *et al*. (2002). In brief: 11–12 mg of xylem powder was extracted in 2 ml distilled water by heating in steam for 30 min. Total NSCs were quantified by incubating 500 μl of the extract with 500 μl of amyloglucosidase (*Aspergillus niger*, 70 U mg^−1^; Sigma‐Aldrich) at 49°C for 15 h to digest starch to glucose. Soluble sugars were quantified from the remaining extract after centrifugation by enzymatic conversion of sucrose (invertase), fructose (phosphoglucose isomerase, PGI), and glucose (glucose‐hexokinase assay) to gluconate‐6‐phosphate. This reaction results in an increase in NADH+H^+^ concentration, which was measured spectrophotometrically at 340 nm using a Multiskan GO Type 1510 plate reader (Thermo Fisher Scientific, Vantaa, Finland). Starch concentration was calculated as total NSC minus soluble sugars. Calibration and quality control used pure standards of starch, glucose, fructose, and sucrose (Sigma‐Aldrich), as well as standard plant powder material (Orchard leaves; Leco, St Joseph, MI, USA).

### Sugar and starch extraction for stable isotope analysis

Stem phloem and xylem tissues were analysed for δ^18^O and δ^2^H values of phloem sugar, xylem sugar, and xylem starch. Phloem starch isotopes were not analysed due to limited sample amounts. For each stem core, phloem and xylem tissues were dried, milled, and extracted for water‐soluble sugars following Lehmann *et al*. (2020). 40 mg of phloem and 170 mg of xylem were extracted in hot water (85°C, 30 min), centrifuged, and purified using ion‐exchange cartridges (OnGuard II H, A, and P, Dionex; Thermo Fisher Scientific, Bremen, Germany). Purified solutions were pipetted into silver capsules (target 0.5–1 mg sugar), freeze‐dried, and sealed.

Xylem starch was extracted following Gleixner *et al*. (1998): after removal of water‐soluble compounds, pellets were washed with a methanol : chloroform : water solution (12 : 3 : 5) and deionised water, gelatinised in hot water, digested with α‐amylase (*Bacillus licheniformis*, 3.0 U ml^−1^) at 85°C for 120 min, and filtered (Vivaspin 6; Sartorius Stedim Biotech GmbH, Göttingen, Germany). Purified digests were pipetted into silver capsules (target 0.5–1 mg sugar), freeze‐dried, and sealed. For beech, 87.1% of samples yielded > 0.5 mg of material. Spruce samples were pooled by campaign due to low starch content.

Isotope values of phloem sugar, xylem sugar, and xylem starch (δ^18^O_PS/XS/XSt_ and δ^2^H_PS/XS/XSt_) were determined by first equilibrating the samples with water of a known δ^2^H value for 2 h (Schuler *et al*., 2022), followed by high‐temperature pyrolysis (PYROcube; Elementar, Hanau, Germany) coupled to an isotope ratio mass spectrometer (IRMS, MAT253; Thermo Fisher Scientific, Bremen, Germany) (Saurer *et al*., 2025). A constant exchangeable hydrogen fraction of 0.3 and a fractionation factor of 1.082 were used to calculate the δ^2^H value of carbon‐bound nonexchangeable hydrogen (Schuler *et al*., 2022). Blank corrections removed enzyme contributions; offset corrections used wheat starch standards (Fluka, Buchs, Switzerland). Analytical precision was 0.2–0.3‰ for δ^18^O and 5–10‰ for δ^2^H. Apparent isotopic fractionation between xylem sugar and xylem starch was calculated as follows: *ε*_XS–XSt_ (‰) = (δ_XS_ − δ_XSt_)/(1 + δ_XSt_/1000).

### Intra‐annual tree‐ring separation, cellulose extraction, and stable isotope analysis

Additional stem cores were collected on 22 November 2022 to ensure completion of xylogenesis. Three trees per species were used to determine intra‐annual α‐cellulose isotope values (δ^18^O_C_ and δ^2^H_C_) for 2021 and 2022. Cores were scanned and ring widths measured. Each annual ring was cut into 50 μm slices along the direction of growth using a rotary microtome (RM2245; Leica Biosystems, Nussloch, Germany). To obtain sufficient material for isotope analysis, slices were pooled into three equal‐width subsamples per ring. α‐cellulose was extracted and analysed following Diao *et al*. (2025). Reported δ^2^H values of sugar, starch, and cellulose represent carbon‐bound, nonexchangeable hydrogen. All isotope values are expressed relative to the international Vienna Standard Mean Ocean Water (VSMOW) scale.

### Modelling the fraction of isotopic exchange during cellulose synthesis

Cellulose is synthesised from glucose moieties derived mainly from leaf‐exported sucrose. The isotopic composition of cellulose (δ_C_) can be modelled using a generalised two‐process model: one fraction (1 − *f*) of O and carbon‐bound H comes from leaf water (δ_LW_) during photosynthetic reactions subject to biosynthetic fractionation (*ε*_A_); the other fraction (*f*) comes from xylem water (δ_XW_), after sucrose export from leaves, including phloem loading and transport, and during postphotosynthetic reactions of cellulose synthesis in the stem cambium, with an associated biosynthetic fractionation (*ε*_H_) (Roden *et al*., 2000). Thus,

(Eqn 1) $$\delta_{C}=\left(1-f\right)\left(\delta_{\mathrm{LW}}\left(1+\varepsilon_{A}\right)+1000\varepsilon_{A}\right)+f\left(\delta_{\mathrm{XW}}\left(1+\varepsilon_{H}\right)+1000\varepsilon_{H}\right)$$

Note that in Eqn 1, the initial sugar substrate from leaves is usually assumed to be sucrose, whose isotope composition is expressed as: δ_LSuc_ = δ_LW_(1 + *ε*_A_) + 1000*ε*_A_.

Rearranging Eqn 1, *f* is expressed as:

(Eqn 2) $$f=\frac{\delta_{C}-\delta_{\mathrm{LW}}\left(1+\varepsilon_{A}\right)-1000\varepsilon_{A}}{\delta_{\mathrm{XW}}\left(1+\varepsilon_{H}\right)-\delta_{\mathrm{LW}}\left(1+\varepsilon_{A}\right)+1000\left(\varepsilon_{H}-\varepsilon_{A}\right)}$$

For oxygen, *ε*_A_ = *ε*_H_ = 0.027 (Sternberg & DeNiro, 1983). For hydrogen, *ε*_A_ = −0.171 and *ε*_H_ = +0.158 (Yakir & DeNiro, 1990). A sensitivity analysis of how 1% and 50% variations in *ε*_A_ and *ε*_H_ around these commonly applied values affect the *f* calculations is provided in Notes S1. Regarding a 1% change in *ε*_A_ and *ε*_H_, the largest effect is caused by *ε*_A_ on *f*_O_; even so, it results in only a 0.0133 change in *f*_O_ (and 0.0024 in *f*_H_). By contrast, a 1% variation in *ε*_H_ produces smaller changes in both *f*_O_ (0.0051) and *f*_H_ (0.0034). Because *ε*_H_ is held constant, the resulting *f* values are interpreted as the fraction of leaf sucrose undergoing isotopic exchange with xylem water along the entire pathway from the leaf to stem cellulose, acknowledging that hydrogen exchange also includes additional nonequilibrium processes. Replacing δ_C_ with δ_PS_ yields an analogous estimate (*f*_O_′) describing isotopic exchange along the pathway of phloem sugar transport between leaves and phloem cells at breast height.

Leaf water isotope composition (δ_LW_) was modelled using the steady state Craig–Gordon equation (Cernusak *et al*., 2016):

(Eqn 3) $$\delta_{\mathrm{LW}}=\left(\varepsilon^{+}+\varepsilon_{k}\right)\left(1-\mathrm{RH}\right)\left(1+\frac{\delta_{\mathrm{XW}}}{1000}\right)+\delta_{\mathrm{XW}}$$

where *ε* ^+^ is the equilibrium isotope fractionation factor between liquid water and vapour and *ε*_k_ is the kinetic isotope fractionation factor during diffusion of water from the leaf intercellular air spaces to the atmosphere. Eqn 3 assumes (1) atmospheric vapour is in isotopic equilibrium with the source water (Szejner *et al*., 2020); (2) canopies are aerodynamically coupled to the ambient environment (Szejner *et al*., 2020); and (3) no Péclet effect (Kahmen *et al*., 2013; Holloway‐Phillips *et al*., 2023). *ε* ^+^ and *ε*_k_ were calculated using the equations provided in Cernusak *et al*. (2016). Leaf temperature (*T*_leaf_) is required to calculate *ε* ^+^, where *T*_leaf_ = *T*_air_ is assumed (Cheesman & Cernusak, 2016; Holloway‐Phillips *et al*., 2023). Stomatal conductance (*g*_s_) and boundary layer conductance (*g*_b_) are required to calculate *ε*_k_. *g*_s_ of spruce was estimated from vapour pressure deficit (VPD) using an empirical equation (*g*_s_ = 173.5e^−0.97VPD^; McDowell *et al*. 2008); the *g*_s_ of beech was taken as 0.06 mol H_2_O m^−2^ s^−1^ higher than that of spruce (Diao *et al*., 2024). *g*_b_ was set to 28 mol H_2_O m^−2^ s^−1^ (Holloway‐Phillips *et al*., 2023). Climate inputs (*T*_air_, RH, and VPD) were calculated as 30‐d rolling daytime averages to reduce day‐to‐day noise, in which daytime was defined as the period between 3 h after sunrise and 3 h before sunset to exclude early morning and late evening periods when photosynthetic activity is generally low and environmental conditions are less representative of active gas exchange. Climate data were obtained from a meteorological station located *c*. 400 m from the study site.

### Data analysis

All statistical analyses were conducted using the R software v.4.4.1 (R Core Team, 2024). Relationships among variables were evaluated using ordinary least squares regression. Two‐sample *t*‐tests were used to assess differences in climate variables between years, isotope compositions among compounds, NSC concentrations between species and years, and *f* values between species and years.

## Results

### Climate, radial growth, and NSC dynamics

**Fig. 1:**
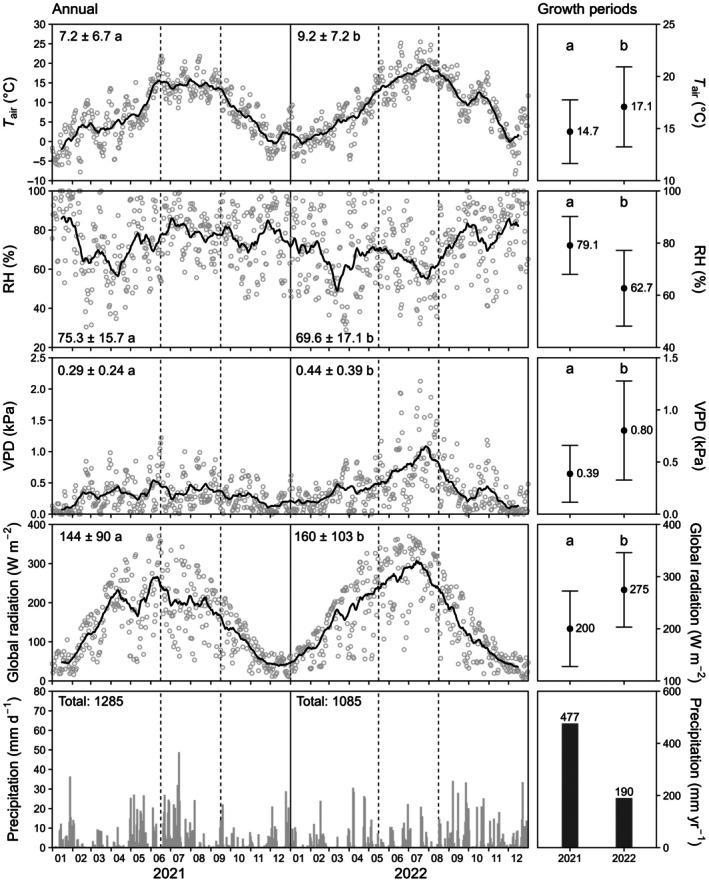
Seasonal and annual climate variability in 2021 and 2022 at the study site. Grey circles and bars show daily gridded climate data (250‐m resolution; Meteotest AG); solid lines represent 30‐d rolling averages. Left panels show values of annual means (±SD) or annual totals for each year (precipitation totals shown in mm). Right panels compare mean (±SD) or total climate conditions during the defined growth periods for 2021 and 2022, marked by vertical dashed lines. Different letters denote significant differences at *P* < 0.05. RH, relative humidity; *T*_air_, air temperature; VPD, vapour pressure deficit.

Annual mean *T*_air_, VPD, and global radiation were significantly higher in 2022 than in 2021, whereas annual mean RH and total precipitation were significantly lower (Fig. 1). The same pattern held during the identified growth periods: 2022 was consistently warmer and drier, and unlike the relatively stable conditions in 2021, 2022 exhibited clear increasing trends in *T*_air_ and VPD and a declining trend in RH. Together, these results indicate that 2022 was warmer and drier than 2021.

**Fig. 2:**
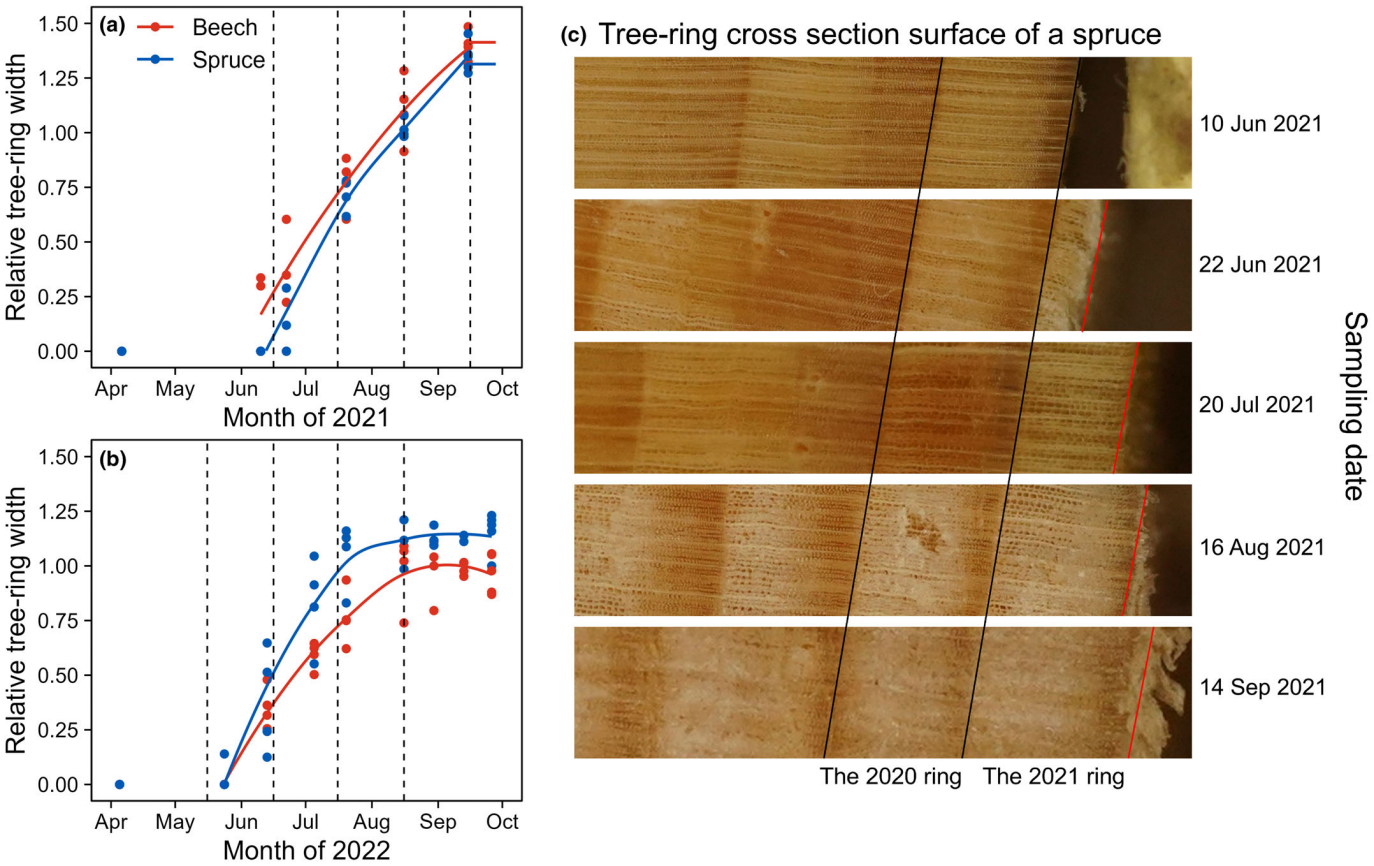
Intra‐annual radial growth of beech and spruce in 2021 and 2022. Relative tree‐ring width on each sampling date in 2021 (a) and 2022 (b), expressed as the ratio of current‐year ring width to the preceding year. Values from the first sampling date in early April are set to zero. In (a), horizontal segments indicate average relative tree‐ring widths measured on cores collected in 2022, representing completed growth of the 2021 ring; these are shown for comparison with the mid‐September 2021 values. Vertical dashed lines divide the growth periods into three equal‐length intervals corresponding to the three segments used for α‐cellulose isotope analysis. Colours distinguish species. (c) Example core images from a spruce tree sampled in 2021. The 2021 ring boundaries are marked in red and the 2020 ring in black. Keeping the 2020 ring width visually constant across cores highlights the progression of radial growth from early to late season.

Intra‐annual radial growth was evaluated using relative tree‐ring width measurements derived from stem core images collected on each sampling date (Fig. 2a,b). An example core image is shown in Fig. 2(c). Based on the ring‐width patterns observed in the intra‐annually sampled cores, we conservatively defined the growth periods for both beech and spruce as June 16 to September 16 in 2021 and May 16 to August 16 in 2022 (Fig. 2a,b). Higher resolution growth monitoring was not available to assess potential differences in growth periods between species. Nevertheless, the seasonal tree‐ring sampling shown in Fig. 2 indicates that the growth periods were similar between species in both 2021 and 2022. Although relative tree‐ring width had not fully plateaued by the final sampling date in mid‐September 2021 (Fig. 2a), measurements of fully completed 2021 rings from samples collected in 2022 (horizontal segments in Fig. 2a) did not differ significantly from those obtained in mid‐September 2021. This supports defining mid‐September as the end of the 2021 growth period. The average tree‐ring width of beech was 0.73 ± 0.39 mm in 2021 and 0.84 ± 0.51 mm in 2022. For spruce, widths were 0.68 ± 0.30 mm and 0.69 ± 0.36 mm, respectively. Although beech showed a 15% increase in mean ring width in 2022, tree‐ring width did not differ significantly between years for either species (*P* = 0.68 and 0.96 for beech and spruce, respectively).

**Fig. 3:**
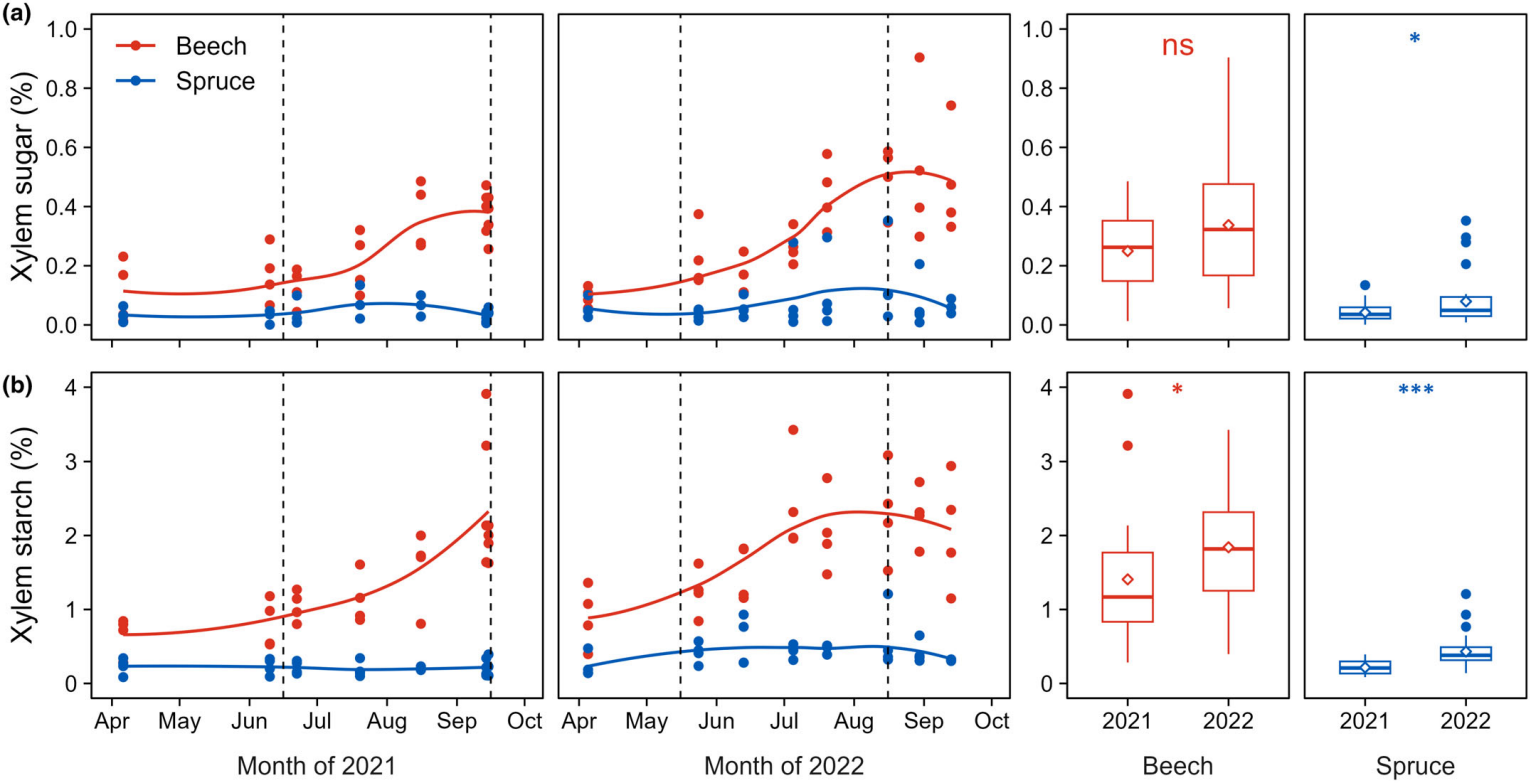
Seasonal variations in xylem sugar (a) and xylem starch (b) concentrations in beech and spruce in 2021 and 2022. Different colours indicate species, and coloured lines show smoothed temporal trends. Vertical dashed lines mark the defined growth periods. Right‐hand panels compare concentrations between 2021 and 2022 for each species using box plots. Diamonds indicate means; dots represent outliers; boxes represents the median and the 25% and 75% quartiles; whiskers represent the 10% and 90% ranges. Significance levels: ns,not significant (*P* > 0.05); *, *P* < 0.05; ***, *P* < 0.001.

In beech, xylem sugar and starch concentrations increased over the course of the growth periods (Fig. 3), whereas spruce maintained significantly lower concentrations of both compounds (*P* < 0.001) with little temporal variation. For both species, growth and NSC concentrations plateaued earlier in 2022. Across species, mean xylem starch concentrations were significantly higher in 2022 than in 2021. In spruce, xylem sugar concentrations were also significantly higher in 2022 than in 2021.

### δ^18^O and δ^2^H of water, sugar, starch, and cellulose

**Fig. 4:**
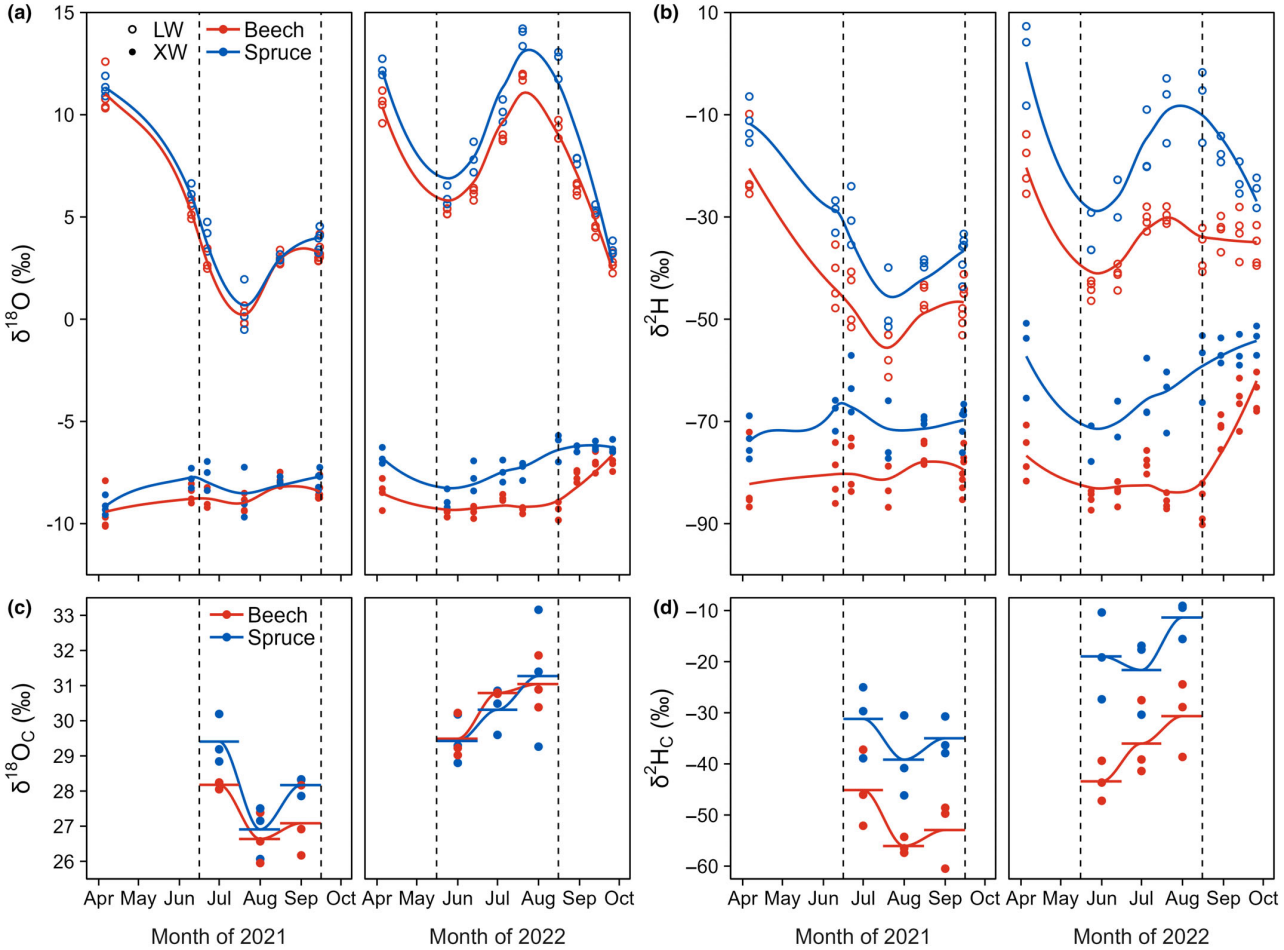
Seasonal variations in the oxygen (δ^18^O) and hydrogen (δ^2^H) isotope compositions of xylem water (δ_XW_, filled circles) and modelled leaf water (δ_LW_, open circles) (a, b), as well as tree‐ring cellulose (δ_C_, c, d) in beech and spruce in 2021 and 2022. Coloured lines indicate smoothed trends for each species. Vertical dashed lines mark the growth periods. δ_LW_ was modelled using Eqn 3. In panels (c) and (d), horizontal coloured lines indicate mean δ_C_ values within each of the three equal‐length intervals in the growth periods.

In spruce, the isotope composition of xylem water (δ_XW_) increased steadily in 2022 but remained relatively stable in 2021, likely reflecting enhanced evaporative isotopic enrichment of shallow soil water under the warmer and drier 2022 conditions (Fig. 4a,b). In beech, δ_XW_ only began to increase after the 2022 growth period, possibly due to deeper water uptake buffering xylem water against early‐season surface evaporation. The δ_XW_ of spruce was systematically higher than that of beech, likely reflecting the shallower rooting system of spruce, which took up isotopically enriched soil water from upper soil layers. Modelled isotope composition of leaf water (δ_LW_), derived from δ_XW_ and climate data (Eqn 3), displayed larger seasonal amplitudes than δ_XW_ and also showed increasing trends in 2022 for both species (Fig. 4a,b).

For the isotope composition of tree‐ring cellulose (δ_C_), three values per year were temporally assigned by dividing the growth period into three equal‐length intervals. δ^18^O_C_ of both species exhibited clear increases in 2022 relative to 2021, whereas an increase in δ^2^H_C_ in 2022 was observed only for beech (Fig. 4c,d). In the remaining cases, δ_C_ first decreased and then increased during the growth periods (Fig. 4c,d).

No distinct temporal patterns were observed in the isotope composition of phloem sugar (δ_PS_), xylem sugar (δ_XS_), and xylem starch (δ_XSt_) across species or years (Fig. S2). However, in beech, δ^18^O_XS_ significantly correlated with xylem sugar concentration, whereas δ^18^O_XSt_ showed no relationship with xylem starch concentration (Fig. S3a,c). Consequently, apparent oxygen isotope fractionation between xylem sugar and starch ($\varepsilon_{\mathrm{XS}-\mathrm{XSt}}^{O}$) was significantly correlated with total xylem NSC concentration, with $\varepsilon_{\mathrm{XS}-\mathrm{XSt}}^{O}$ values closer to zero occurring at higher NSC concentrations (Fig. S3e). No analogous hydrogen isotope relationships were observed (Fig. S3).

**Fig. 5:**
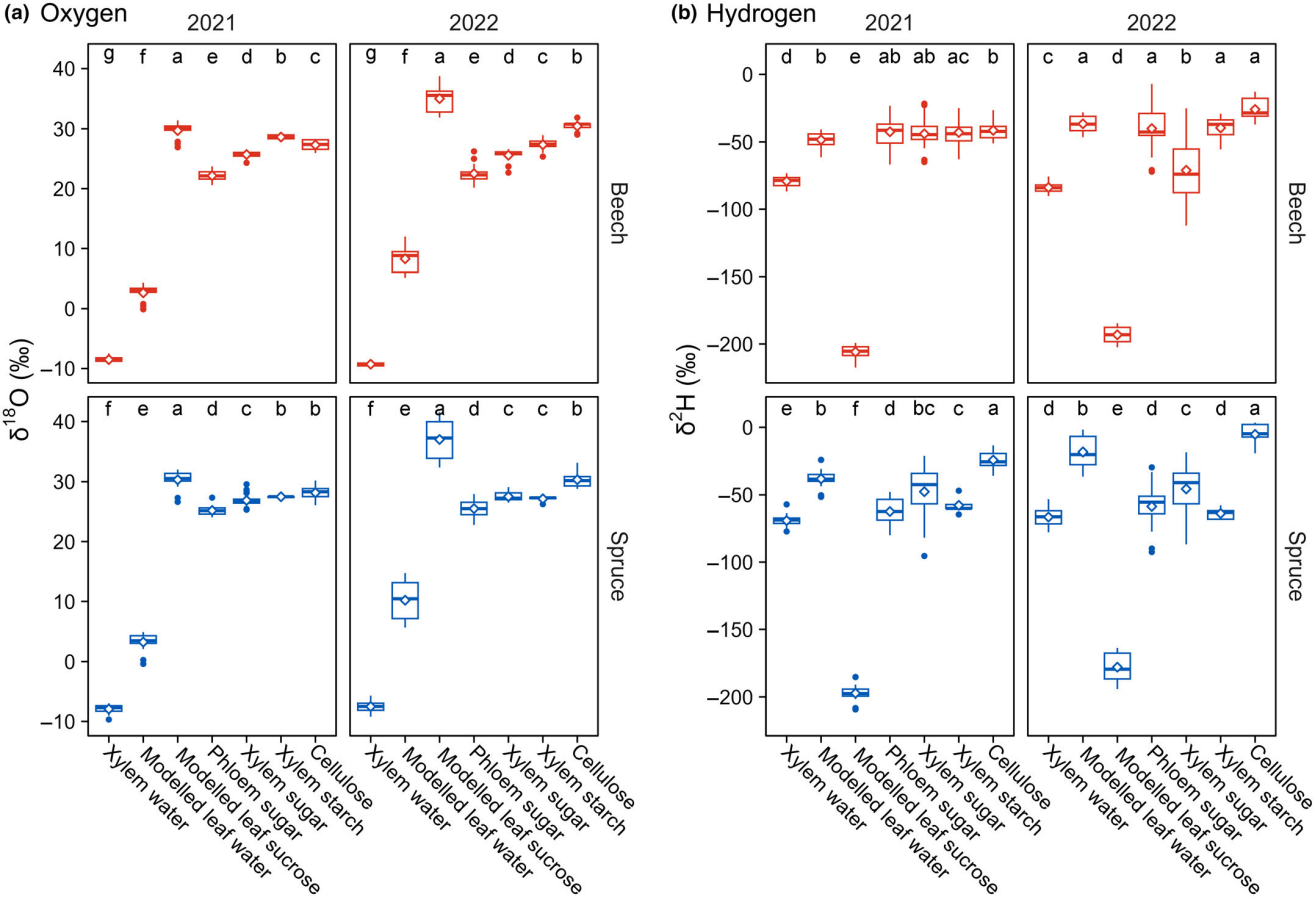
Oxygen (δ^18^O, a) and hydrogen (δ^2^H, b) isotope compositions of xylem water, modelled leaf water, modelled leaf sucrose, phloem sugar, xylem sugar, xylem starch, and cellulose in beech and spruce during the 2021 and 2022 growth periods. Modelled values for leaf water and leaf sucrose were derived using Eqns (Eqn 1), (Eqn 2), (Eqn 3). Only measurements within the defined growth periods (16 June 2021 to 16 September 2021 and 16 May 2022 to 16 August 2022) are shown. Different colours indicate species. Diamonds represent means; dots represent outliers; boxes show the median and the 25% and 75% quartiles; whiskers represent the 10% and 90% ranges. Different letters indicate significant differences (*P* < 0.05) among compounds within each species and year.

Across the growth periods of both years, δ^18^O_XSt_ was significantly higher than δ^18^O_PS_ in both species (*P* < 0.001), whereas δ^2^H_XSt_ did not differ significantly from δ^2^H_PS_ in beech (Fig. 5). Additionally, δ^18^O_XSt_ was higher than δ^18^O_XS_ in beech in both years (*P* < 0.001), but not in spruce in 2022. For hydrogen, δ^2^H_XSt_ exceeded δ^2^H_XS_ in beech but was significantly lower than δ^2^H_XS_ in spruce in 2022 (*P* < 0.01). In general, δ_PS_ and δ_XS_ were lower than δ_C_, except for δ^2^H_PS_ and δ^2^H_XS_ of beech.

**Fig. 6:**
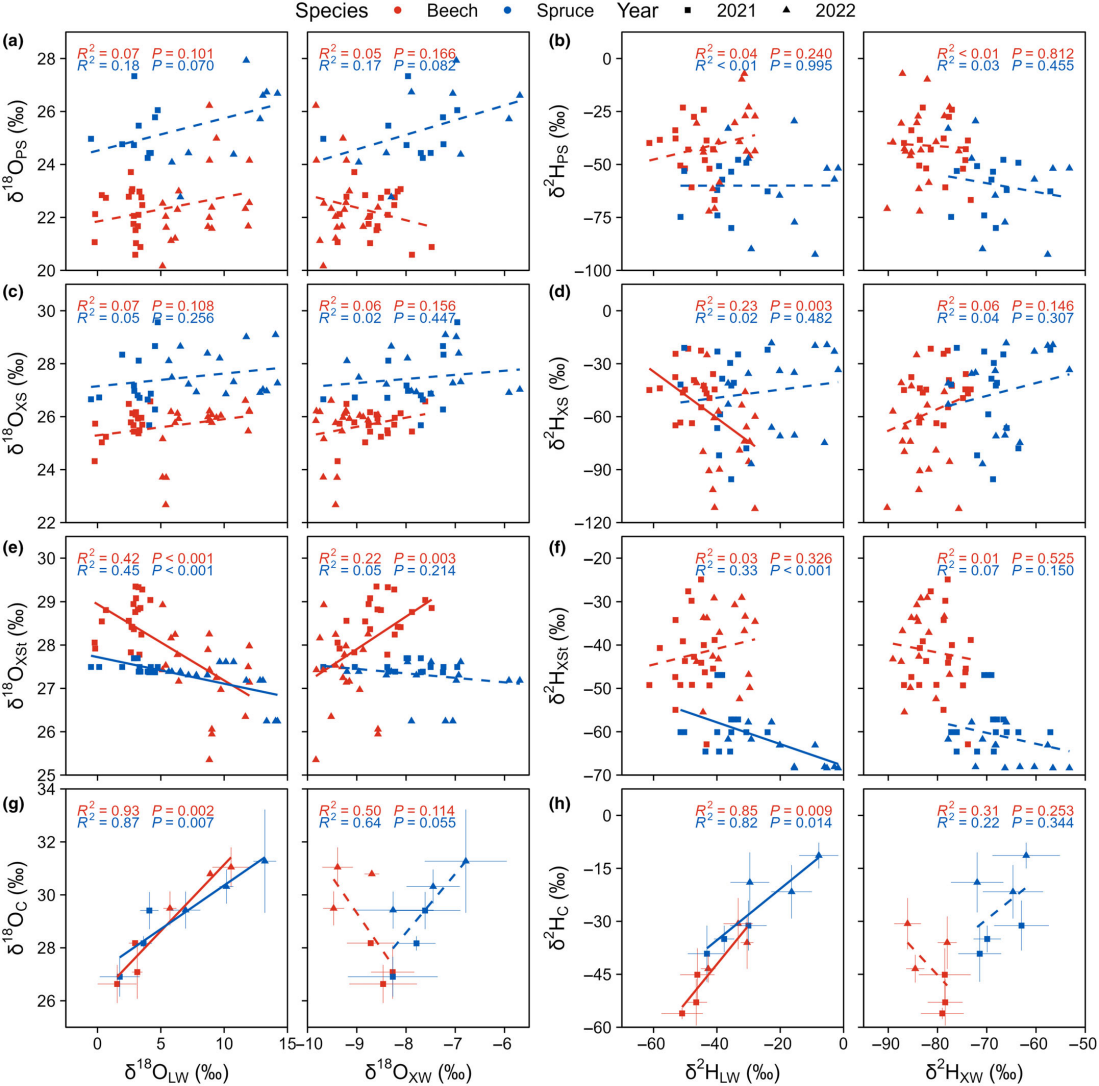
Relationships between the oxygen (δ^18^O) and hydrogen (δ^2^H) isotope compositions of phloem sugar (δ_PS_, a, b), xylem sugar (δ_XS_, c, d), xylem starch (δ_XSt_;, e, f), and cellulose (δ_C_, g, h) versus modelled leaf water (δ_LW_) and measured xylem water (δ_XW_) in beech and spruce during the 2021 and 2022 growth periods. Different colours indicate species; different symbols indicate years. Solid lines denote significant (*P* < 0.05) linear relationships fitted across years for each species; dashed lines denote nonsignificant relationships. Regression statistics (*P*values and *R* ^2^) are provided.

Neither δ_LW_ nor δ_XW_ showed consistent positive relationships with δ_PS_, δ_XS_, or δ_XSt_ in either species (Fig. 6a–f), with the exception of δ^18^O_XW_ vs δ^18^O_XSt_ in beech (*R* ^2^ = 0.22, *P* = 0.003; Fig. 6e). Similarly, δ_PS_, δ_XS_, and δ_XSt_ were not positively correlated with δ_C_ (Fig. S4). By contrast, δ_C_ showed seasonal variations similar to modelled δ_LW_ (Fig. 4), reflected in strong positive relationships between δ_C_ and δ_LW_ but not δ_XW_ for both oxygen and hydrogen (Fig. 6g,h). These stronger δ_C_–δ_LW_ correlations held between and within years for beech (Fig. S5). For beech, δ_C_ was not positively related to δ_XW_, whereas δ_C_–δ_LW_ correlations were consistently significant. Relationships were generally stronger for oxygen than for hydrogen, indicated by higher *R* ^2^ values across years (Fig. 6g,h) and within individual years, except for δ_C_ vs δ_LW_ in 2021 in both species (Fig. S5).

Modelled leaf sucrose (δ_LSuc_) showed significantly higher δ^18^O and significantly lower δ^2^H than both δ_XW_ and δ_C_ (*P* < 0.001; Fig. 5). The δ_LSuc_ values also differed markedly from measured δ_PS_ and δ_XS_ in both species and years (*P* < 0.001), indicating that stem‐derived phloem and xylem sugars do not isotopically correspond to newly assimilated sugars before postphotosynthetic modification.

### Comparison of isotopic exchange between oxygen and hydrogen

**Fig. 7:**
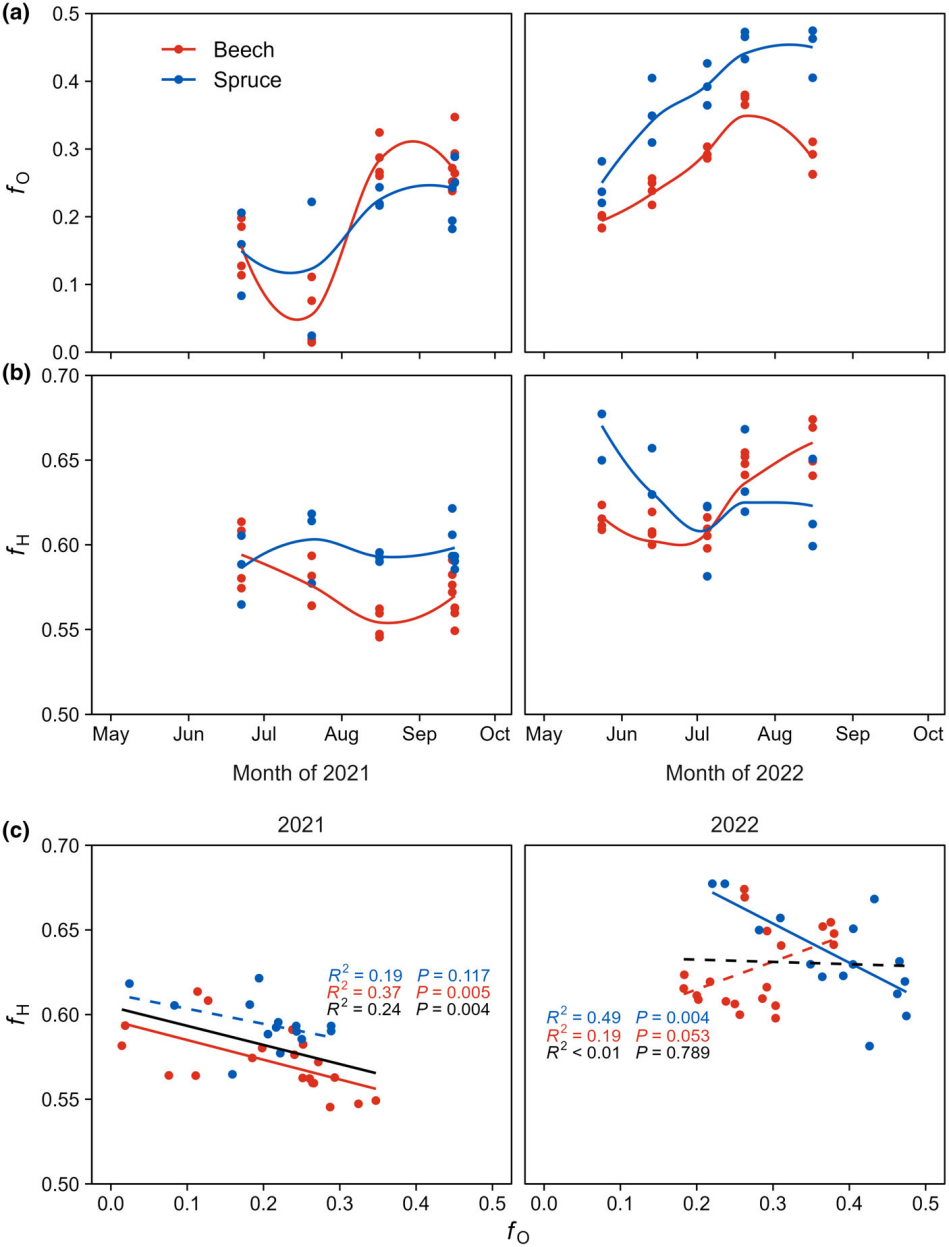
Seasonal variations in the fractions of oxygen (*f*_O_; a) and hydrogen (*f*_H_; b) isotopic modification and their interrelationship (c) in beech and spruce in 2021 and 2022. Different colours indicate species. In panel (c), solid coloured lines represent significant (*P* < 0.05) relationships within species; the solid black line denotes significant (*P* < 0.05) relationships across species; dashed lines indicate nonsignificant relationships. Regression statistics (*P*‐values and *R* ^2^) are provided.

Because of the lack of congruence between δ_PS_ and the modelled δ_LSuc_ (Fig. 5), we used modelled δ_LSuc_ for estimating *f* values, in order to more accurately capture the isotope signal of fresh assimilates required to apply Eqn 3 (Fig. 7). It should also be noted that δ_C_ was measured at a lower temporal resolution than the δ_XW_ and δ_LSuc_ data. Therefore, δ_C_ was assumed to be constant within each of the three equal‐length growth period intervals for the purpose of estimating *f*. These constant δ_C_ values correspond to the mean δ_C_ values of the three equal‐width subsamples per ring (see the horizontal coloured lines in Fig. 4c,d).

Across species, *f*_O_ ranged 0.01–0.47, whereas *f*_H_ ranged 0.55–0.68; both were significantly higher in 2022 than in 2021 (*P* < 0.001; Fig. 7a,b). The fraction *f*_O_ increased over the growth period in both years and both species, while *f*_H_ showed no comparable temporal trend. In 2021, *f*_O_ and *f*_H_ were significantly negatively correlated in beech and across species (Fig. 7c), but these relationships weakened markedly in 2022, remaining significant only in spruce.

**Fig. 8:**
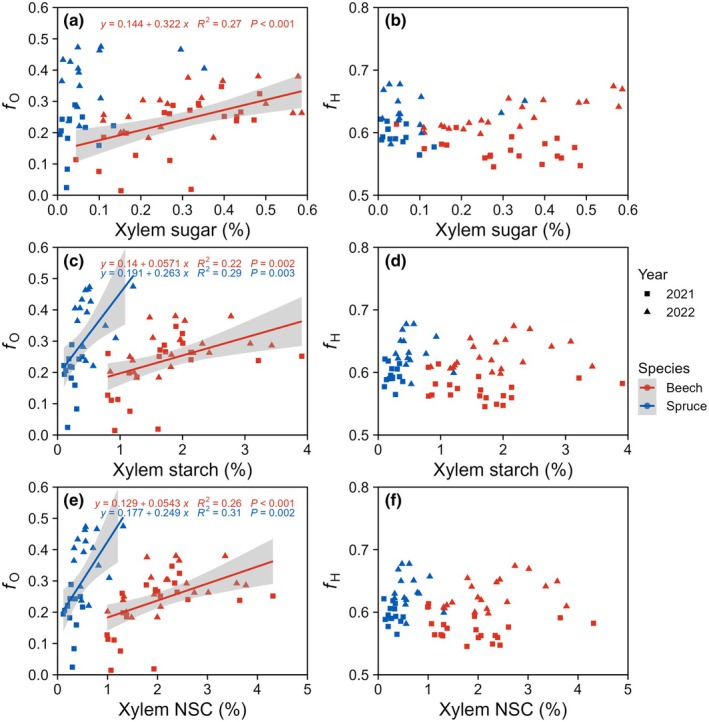
Relationships between isotopic modification fractions (*f*) and concentrations of xylem sugar (a, b), starch (c, d), and total xylem nonstructural carbohydrate (e, f) in beech and spruce in 2021 and 2022. Different colours indicate species; different symbols indicate years. Solid lines with shaded 95% confidence intervals represent significant (*P* < 0.05) linear relationships fitted across years for each species. Regression equations, *P*‐values, and *R* ^2^ are provided for significant relationships only.

Across both years, *f*_O_ was positively correlated with xylem sugar, starch, and total NSC concentrations in both species, whereas *f*_H_ showed no such relationships (Fig. 8). Both *f*_O_ and *f*_H_ were significantly positively related to *T*_air_, VPD, and RH, except for spruce *f*_H_ in 2022 (Fig. S6). Correlations between *f*_O_ with climate variables (*R* ^2^ = 0.34–0.78; Fig. S6a,c,e) were generally stronger than correlations with NSC (*R* ^2^ = 0.22–0.31; Fig. 8a,c,e).

When analysed separately by year, *f*_O_ was significantly correlated with VPD in both species in 2022, whereas *f*_H_ was correlated with VPD only in beech (Fig. S7a,b). No significant relationships with VPD were found in 2021. Additionally, *f*_O_ was correlated with xylem NSC concentration in beech in both years, but not in spruce, while *f*_H_ was significantly correlated with NSC only in beech in 2022 (Fig. S7c,d).

## Discussion

### Intra‐annual dynamics of stem sugar and starch concentrations do not directly influence their oxygen and hydrogen isotopic signals

Sugars and starch in the stem are considered precursors for tree‐ring cellulose synthesis. Temporal changes in their concentrations – reflecting shifts in the balance of carbon fluxes into and out of these pools – are expected to affect both their isotopic composition and their relative contribution to cellulose formation, thereby shaping the isotopic signals ultimately recorded in tree‐ring cellulose. Towards the end of the growth period, the concurrent increases in soluble sugars and starch in beech stems (Fig. 3) suggest partitioning to NSC storage and a source‐dominated state in which carbon supply exceeds sink demand for growth and maintenance. This stored carbon can then support growth the following spring (Hoch *et al*., 2003). Although both species had higher NSC contents in 2022 than in 2021 (Fig. 3), only beech showed a substantial increase in tree‐ring growth. This suggests that warmer conditions in 2022 increased carbon availability sufficiently in beech to support both storage and growth. In spruce, carbon assimilation likely also exceeded growth demand, but growth was apparently limited by sink activity or environmental constraints, especially given the drier conditions in 2022.

Despite these seasonal shifts in sugar and starch concentrations, their oxygen and hydrogen isotope compositions generally did not covary with concentration changes (Figs 3, S2, S3), contrary to Hypothesis 1. One exception was a significant positive relationship between xylem sugar concentration and δ^18^O_XS_ across years in beech (Fig. S3a). Correspondingly, although δ^18^O_XSt_ was on average higher than δ^18^O_XS_ across years in beech (Fig. 5), their difference ($\varepsilon_{\mathrm{XS}-\mathrm{XSt}}^{O}$) approached zero as xylem NSC concentration increased (Fig. S3e). This pattern cannot be explained by seasonal variation in leaf or xylem water isotopes, as no clear relationships were found for δ^18^O_XS_ or δ^18^O_XSt_ with δ^18^O_LW_ or δ^18^O_XW_ (Fig. 6). Instead, it may reflect a rising proportion of sucrose, which is typically more ^18^O‐enriched than hexoses (Lehmann *et al*., 2017), within the bulk sugar pool as sugar concentrations increase through the growing season.

### Intra‐annual isotopic variations in tree‐ring cellulose are driven primarily by water signals, not by sugar precursors

We found that although a significant portion of the original leaf isotopic signal was modified before cellulose synthesis (*f*_O_ < 0.47; *f*_H_ = 0.55–0.68; Fig. 7), δ_C_ correlated strongly with modelled δ_LW_ rather than measured δ_XW_ for both oxygen and hydrogen in beech (Figs 6g,h, S5). This likely reflects the limited seasonal variability of δ_XW_ relative to δ_LW_ (Fig. 4a,b), such that even extensive exchange with xylem water could not override the larger seasonal range of δ_LW_ that becomes imprinted in cellulose. For spruce, δ_LW_ and δ_XW_ were similarly reflected in δ_C_ at the intra‐annual scale, especially for δ^18^O (Fig. S5). These findings contrast with previous intra‐annual studies reporting stronger imprinting of xylem water than leaf water on tree‐ring δ^18^O (Offermann *et al*., 2011; Treydte *et al*., 2014; Martínez‐Sancho *et al*., 2023). There is evidence that oxygen atom exchange between water and carbonyl oxygen is not constant and depends on NSC turnover times (Song *et al*., 2014). Thus, differences in source‐sink relationships affecting NSC turnover might be a reason for the differences observed among studies. We also found that δ^18^O and δ^2^H in leaf or xylem water were similarly captured in cellulose, contrary to Hypothesis 1.

We found no direct isotopic relationship between tree‐ring cellulose and stem sugars or starch (Fig. S4), in contrast to studies in which close isotopic coupling between leaf sugars and leaf cellulose has been observed in herbaceous plants (Lehmann *et al*., 2017; Holloway‐Phillips *et al*., 2022). This decoupling in trees likely reflects the longer turnover times and greater mixing among sugar pools of different ages in large woody tissues (Richardson *et al*., 2015; Hartmann & Trumbore, 2016), which dilute seasonal isotopic signals of specific sugar pools in tree‐ring cellulose (Szejner *et al*., 2025). Seasonally variable isotopic exchange between phloem sugar and xylem water at the site of cellulose synthesis likely further weakens the relationship between δ_PS_ and δ_C_. It should be noted that several significant negative relationships were observed for δ^18^O_C_–δ^18^O_XSt_ and δ^2^H_C_–δ^2^H_XS_ in beech, as well as for δ^2^H_C_–δ^2^H_XSt_ in spruce (Fig. S4). Nevertheless, these negative relationships were not consistent across species or between elements, and most of the tested relationships between cellulose and sugar or starch remained nonsignificant. Further studies are needed to clarify the mechanisms underlying these occasional negative correlations and to determine how environmental and physiological factors modulate the transfer of isotopic signals from NSC to cellulose, including instances where the relationship shifts from positive to negative.

Interestingly, δ^2^H signatures did not differ between xylem starch and phloem sugars in either species, and xylem starch was even ^2^H‐depleted relative to xylem sugar in spruce (Fig. 5). This does not support the expectation that starch should be ^2^H‐enriched relative to sugars (Cormier *et al*., 2018; Angove *et al*., 2025). The mechanism by which starch becomes ^2^H‐enriched relative to sugars is not clear. Potentially, the oxidative pentose phosphate pathway (OPPP), which operates in leucoplasts, could ^2^H‐enrich position H‐1 of glucose derivatives relative to G6P in the cytosol. However, the OPPP also operates in the cytosol, in which case, G6P, which is the substrate for cellulose synthesis, could be directly impacted (Fig. S1). Alternatively, the conditions under which starch utilisation is increased may be related to shifts in other reactions such as PGI (Wieloch *et al*., 2025). Furthermore, δ^2^H_XSt_ did not differ significantly between years in beech but was lower in 2022 than in 2021 in spruce, whereas δ^2^H_C_ was higher in 2022 than 2021 in both species (Fig. 5). These observations do not support the idea that drought‐ or defoliation‐driven ^2^H‐enrichment in cellulose arises from increased utilisation of starch reserves (Nabeshima *et al*., 2018; Lehmann *et al*., 2024a). Instead, ^2^H enrichment may reflect shifts in metabolic fluxes affecting glucose precursors in sink tissue rather than the utilisation of ^2^H‐enriched starch (Holloway‐Phillips *et al*., 2026).

### Most isotopic exchange with water occurs during phloem sugar transport

Previous work in oxygen stable isotopes suggests that isotopic exchange can occur before sugars reach the site of cellulose synthesis, such as during phloem loading and transport. Evidence includes oxygen isotope decoupling between phloem and leaf sugars, interpreted as exchange of phloem sugars with less enriched water during phloem loading and transport (Offermann *et al*., 2011; Gessler *et al*., 2013; Treydte *et al*., 2014; Fiorella *et al*., 2022; Pan *et al*., 2024). In line with this, our δ^18^O_PS_ values were lower than modelled δ^18^O_LSuc_. By comparison, δ^2^H_PS_ values were 141‰ higher than modelled δ^2^H_LSuc_ (Fig. 5b). Thus, both oxygen and hydrogen isotopes indicate that phloem sugars become isotopically altered well before reaching the site of cellulose synthesis.

Although bark photosynthesis has been proposed as a possible explanation for low δ^18^O of phloem sugars in twigs (Gessler *et al*., 2013), our δ^2^H data do not support this. Because hydrogen isotope fractionation during photosynthesis is negative (*ε*_A_ ≈ −171‰), photosynthesis of green bark should lead to ^2^H‐depleted phloem sugars relative to xylem water, not to the ^2^H enrichment we observed (Fig. 5b). Although species‐ and compound‐specific *ε*_A_ values have been reported (Holloway‐Phillips *et al*., 2022; Schuler *et al*., 2023), δ^2^H values of bark assimilates would still be lower than δ^2^H_XW_ and therefore cannot explain the observed ^2^H enrichment in phloem sugars.

By substituting δ^18^O_C_ with δ^18^O_PS_ in Eqn 2, we estimated the fraction of oxygen isotopic exchange with xylem water that has occurred between leaves and stem phloem at breast height (*f*_O_′). Mean *f*_O_′ values were 0.67 (beech) and 0.56 (spruce). The *f*_O_′ of beech is comparable to that obtained from phloem sugars in the branch of five tree species (0.70) in the recalculation of the Gessler *et al*. (2013) dataset by Pan *et al*. (2024). In an additional experiment with three tree species, Pan *et al*. (2024), found a substantially lower species‐averaged *f*_O_′ from petiole phloem sucrose (0.25). This likely implies that a longer transport pathway (i.e. branches vs petioles) leads to greater opportunity for oxygen exchange. Because our stem phloem‐derived *f*_O_′ values are comparable to branch phloem values, we suggest that most oxygen exchange in our study already occurred within the canopy (from leaf to petiole to branch) rather than during phloem translocation in the main trunk.

However, the observation that δ^18^O_PS_ < δ^18^O_C_ (Fig. 5) implies unrealistically greater isotopic exchange during leaf‐to‐stem transport than throughout the entire path to cellulose synthesis (i.e. calculated *f*_O_′ > *f*_O_ based on Eqn 2). While direct isotopic comparison between phloem sugar and tree‐ring cellulose is scarce, similar δ^18^O_PS_ < δ^18^O_C_ patterns have been reported in previous studies that investigated phloem sugars in twigs (Treydte *et al*., 2014; Fiorella *et al*., 2022), though not in Szejner *et al*. (2025). Treydte *et al*. (2014) proposed that twig phloem, unlike stem phloem, may not fully represent the whole crown, leaving open the possibility that stem δ^18^O_PS_ > twig δ^18^O_PS_ and thus stem δ^18^O_PS_ > δ^18^O_C_. However, our results based on stem phloem (i.e. δ^18^O_PS_ < δ^18^O_C_) do not support this explanation. Alternatively, the ^18^O‐enrichment of cellulose relative to phloem sugars may suggest unaccounted‐for KIEs during sugar metabolism and/or cellulose polymerisation.

While isotopic exchange with water in carbonyl oxygen is the dominant process by which isotope fractionation occurs, KIEs are also possible. Oxygen KIEs in enzymatic reactions arise when oxygen atoms are involved in bond cleavage or formation, with the largest effects occurring at the bond being broken. For C–O bonds such as glycosidic linkages, primary ^18^O isotope effects at the leaving oxygen can be substantial (*k* ^16^/*k* ^18^ ≈ 1.036 − 1.047; Rosenberg & Kirsch (1981), measured in model glycosidase systems), but this effect is expressed on the oxygen that leaves the molecule and is therefore not retained in the sugar product. By contrast, isotope effects on the oxygens that remain in the sugar are likely much smaller, consistent with general kinetic isotope theory (Cleland, 1982). This applies to key steps in plant carbohydrate metabolism, including the reaction catalysed by sucrose synthase, carbon rearrangements of aldolase, and glycosyl transfer during cellulose synthesis, all of which involve C–O bond cleavage or formation but do not retain the oxygen atoms directly involved in bond breaking. Consequently, only small secondary isotope effects likely influence cellulose δ^18^O, and thus are unlikely to explain the observed enrichment of cellulose relative to phloem sugars without additional processes such as incomplete exchange with water.

It is worth noting that the bulk phloem sugar analysed in this study represents a possible mixture of mainly sucrose, hexoses, raffinose, and sugar alcohols extracted from dried phloem tissue. Although individual sugar compounds differ in their isotope signatures (Schmidt *et al*., 2001; Lehmann *et al*., 2017; Cormier *et al*., 2018), sucrose is typically the dominant (*c*. 95%) compound in phloem exudates of both beech (Gessler *et al*., 2013) and spruce (Gall *et al*., 2002). If this applies to our case, the chemical composition of phloem sugar is unlikely to explain the isotopic uncoupling from leaf sucrose (Gessler *et al*., 2013). However, it is important to acknowledge that the extraction method (from phloem sap vs bulk phloem tissue) may influence the relative composition of sugar compounds. For example, Fink *et al*. (2018) reported that the sucrose concentration in the phloem sap was approximately five times higher than in the mesophyll cell cytosol in beech. In addition, studies have found that sucrose accounts for < 50% of total soluble sugars extracted from bulk phloem tissue in spruce (Lintunen *et al*., 2016; Basile *et al*., 2024). These findings suggest that sucrose may be underrepresented in our extracted bulk phloem sugars, especially for spruce. In the absence of published information on compound‐specific δ^2^H of phloem sugar, we cannot exclude that differing δ^2^H among sugar compounds contribute to observed ^2^H enrichment. However, it is very likely that the δ^2^H of sugar compounds differs due to the large and variable KIEs associated with sugar metabolism (Leadlay *et al*., 1976; Hermes & Cleland, 1984; Canellas & Cleland, 1991; Holloway‐Phillips *et al*., 2025).

### Negative covariation between *f*_O_ and *f*_H_ within growth periods

Including hydrogen alongside oxygen isotopes allowed us to examine covariation between *f*_O_ and *f*_H_ and its implication for hydrologic signal transfer to cellulose for each element. We observed contrasting seasonal dynamics of *f*_O_ and *f*_H_, producing a negative correlation between them in beech in 2021 and in spruce in 2022, but no covariation for beech in 2022 and for spruce in 2021 (Fig. 7). Consistent with Hypothesis 2, this contrasts with previous findings of positive covariation (Luo & Sternberg, 1992) or no covariation (Holloway‐Phillips *et al*., 2022). These earlier studies focused on herbaceous species under controlled conditions, whereas our study is the first to examine *f*_O_–*f*_H_ relationships in tree‐ring cellulose of mature trees in the field. Although this study focused on beech and spruce, the mixed results regarding the covariation between *f*_O_ and *f*_H_ across species and years suggest that a stable *f*_O_–*f*_H_ relationship may not apply universally across different tree species.

Under the cellulose isotope model (Eqn 1), if *ε*_A_, *ε*_H_, δ_LW_, and δ_XW_ remain constant, negative *f*_O_–*f*_H_ covariation would generate a positive correlation between δ^18^O_C_ and δ^2^H_C_, which aligns with our observations (Fig. S8). However, δ_LW_ and δ_XW_ vary widely at seasonal scales (Fig. 4), meaning that seasonal variability in *f*_O_ and *f*_H_ covariation may decouple climatic signals recorded by oxygen and hydrogen. Such decoupling aligns with the commonly observed loss of δ^18^O–δ^2^H covariance from source water to tree‐ring cellulose across years and sites (Holloway‐Phillips *et al*., 2023; Charlet de Sauvage *et al*., 2025; Diao *et al*., 2025).

Although *f*‐value dynamics have been linked to environmental conditions and C‐metabolism processes regulating growth (Song *et al*., 2014; Nabeshima *et al*., 2018; Szejner *et al*., 2020; Holloway‐Phillips *et al*., 2022, 2023; Martínez‐Sancho *et al*., 2023), we did not find opposing effects of climate or NSC on *f*_O_ vs *f*_H_ that would explain their negative correlation (Figs 8, S6, and S7). Instead, differing sensitivities of *f*_O_ and *f*_H_ to climate and NSC in different years likely contributed to the loss of covariation (Fig. 7c). We discuss these relationships further later.

### Climate and NSC drivers of *f* ‐value variability differ between oxygen and hydrogen

We found that *f*_O_ increased both within growth periods and between years (Fig. 7a), corresponding to progressively warmer and drier conditions through the season and to overall drier conditions in 2022 (Figs 1 and 3). The relationships between *f*_O_ and climate variables were driven largely by the greater climate variability in 2022 (Figs S6 and S7), suggesting that seasonal aridity contributes to variation in *f*_O_. Independent evidence supports this: across aridity gradients in northern Australia, *f*_O_ was negatively related to mean annual precipitation (Holloway‐Phillips *et al*., 2023), and seasonal *f*_O_ of *Larix decidua* L. increased with VPD in the Swiss Alps (Martínez‐Sancho *et al*., 2023). We propose that increasing aridity and the associated slowdown in carbon use for growth (Figs 1, 3) increase both the likelihood of sugar loading/unloading during phloem transport and the longer residence time of sugars within sink cells, thereby enhancing opportunities for carbonyl‐oxygen exchange with water (Hill *et al*., 1995; Barbour & Farquhar, 2000; Song *et al*., 2014).

Across both years, *f*_O_ but not *f*_H_ correlated with NSC contents (Fig. 8), supporting Hypothesis 3 that oxygen‐ and hydrogen‐exchange processes differ in their linkage to NSC dynamics. This likely reflects tighter coupling between oxygen‐exchange processes and NSC metabolism. Thus, we suggest that metabolism‐induced variations in isotopic exchange during cellulose synthesis need to be considered, especially for oxygen. Hydrogen‐exchange processes involve large KIEs (Leadlay *et al*., 1976; Hermes & Cleland, 1984; Canellas & Cleland, 1991; Holloway‐Phillips *et al*., 2025), which may vary under physiologically relevant conditions and in response to environmental conditions (Schleucher, 1998; Ehlers *et al*., 2015; Wieloch *et al*., 2022) but are not explicitly incorporated into current cellulose isotope models (Yakir & DeNiro, 1990; Barbour & Farquhar, 2000; Roden *et al*., 2000). Assuming constant biosynthetic isotope fractionation factors (*ε*_A_ and *ε*_H_) means that any variability in isotope fractionation terms becomes absorbed into calculated *f* values. However, sensitivity analyses (Notes S1) suggest that reasonable variation in *ε*_A_ and *ε*_H_ produces only marginal changes in *f* values, particularly for hydrogen.

Nevertheless, a limitation of our study is that δ_LW_ was estimated using the Craig–Gordon model rather than measured directly. Although this approach is widely used, violations of the key assumptions of the model may affect the interpretation of the calculated *f* values. Under ideal conditions, direct measurements of the isotope composition of atmospheric vapour and *T*_leaf_ would help improve the modelling of δ_LW_ (Cernusak *et al*., 2016). In addition, better characterization of the leaf hydraulic design of the studied species would allow a more realistic representation of the presence or absence of a Péclet effect and its relative importance in beech and spruce when modelling δ_LW_ (Zwieniecki *et al*., 2007; Barbour *et al*., 2021, 2024). Moreover, although evidence is still limited, biochemical isotopic fractionation is believed not to be constant (Sternberg & Ellsworth, 2011; Schuler *et al*., 2025; Holloway‐Phillips *et al*., 2026), suggesting that variable *ε*_A_ and *ε*_H_ values should be considered in applications of the cellulose isotope model in which possible. Furthermore, future studies should aim to characterise NSC dynamics and their isotopic signals during the dormant season, as sugars may undergo isotopic exchange with water during this period and subsequently influence the isotopic signature of cellulose formed in the following year. This may be particularly relevant for species that rely on stored NSC from the previous year at the beginning of the growing season (Helle & Schleser, 2004).

### Conclusions

The isotope composition of tree‐ring cellulose is commonly interpreted as reflecting a mixture of leaf and xylem water signals, each carrying distinct climatic information, with their relative influence mediated by the fraction of sugars undergoing isotopic modification downstream of leaves (the *f* value). Using intra‐annual measurements of oxygen and hydrogen isotopes in beech and spruce, we show that, at our site, seasonal variations in leaf water isotope composition are a major driver of the intra‐annual isotopic signal in cellulose, particularly in beech. Along the isotopic pathway from leaf sugars to tree‐ring cellulose, we neither observed a significant influence of water sources on stem sugars and starch nor an influence of stem sugars and starch dynamics on cellulose, suggesting that the isotopic signal transfer from leaves is hampered by mixing processes among stem sugar pools of different ages. Furthermore, we can rule out bark photosynthesis as a major contributor of the observed strong isotopic decoupling of phloem sugars from freshly assimilated leaf compounds. Our results instead suggest that variations in leaf water signals recorded by stem cellulose isotopes are modified during sugar metabolism at the site of cellulose synthesis. As such, the observed negative or absent covariation between *f*_O_ and *f*_H_ within growth periods suggests major differences in the mechanistic processes driving oxygen and hydrogen isotopic patterns in tree‐ring cellulose. We show that the observed seasonal divergence between *f*_O_ and *f*_H_ is likely driven by differential responses to climate and/or NSC dynamics, while the consistent association between stem‐xylem NSC concentrations and *f*_O_ suggests sugar turnover as a major driver of oxygen, but not hydrogen, isotopic modification. Taken together, this study helps refine interpretations of tree‐ring cellulose isotope records by clarifying how isotopic modification post‐sugar‐export from leaves influences the transfer of environmental signals into cellulose, thereby facilitating tree‐ring cellulose isotope‐based reconstructions of forest climate and ecohydrological conditions.

## Competing interests

None declared.

## Author contributions

HD, MH‐P, KT, MS, GA, AG, KM and MML conceived the study. HD, FB, PW, KT, MS, GA and KM developed the methodology. HD, FB, PW and KM collected the data. HD, MH‐P, XS and MML analysed and visualised the data. HD wrote the manuscript, while MH‐P, XS, FB, PW, KT, MS, GA, AG, KM and MML contributed critically to the manuscript.

## Disclaimer

The New Phytologist Foundation remains neutral with regard to jurisdictional claims in maps and in any institutional affiliations.

## Supporting information


**Dataset S1** The dataset analysed in this study.

**Fig. S1** Simplified schematic of major reactions associated with isotope effects during heterotrophic metabolism in the sink‐cell cytosol.
**Fig. S2** Seasonal variations in δ^18^O and δ^2^H of phloem sugar, xylem sugar, and xylem starch in beech and spruce in 2021 and 2022.
**Fig. S3** Relationships between NSC concentration and isotope composition for xylem sugar and xylem starch in beech and spruce in 2021 and 2022.
**Fig. S4** Relationships between tree‐ring cellulose and phloem sugar, xylem sugar, and xylem starch for δ^18^O and δ^2^H isotope compositions in beech and spruce across 2021 and 2022.
**Fig. S5** Relationships between tree‐ring cellulose isotope composition and modelled leaf water or measured xylem water for δ^18^O and δ^2^H in beech and spruce during the 2021 and 2022 growth periods.
**Fig. S6** Relationships between the fraction of isotopic exchange and *T*_ai_, VPD, and RH in beech and spruce during the 2021 and 2022 growth periods.
**Fig. S7** Relationships between the fraction of isotopic exchange and VPD or xylem NSC concentration in beech and spruce during the 2021 and 2022 growth periods.
**Fig. S8** Relationships between δ^18^O and δ^2^H of xylem water, leaf water, and tree‐ring cellulose in beech and spruce during the 2021 and 2022 growth periods.
**Notes S1** Sensitivity analysis.
Please note: Wiley is not responsible for the content or functionality of any Supporting Information supplied by the authors. Any queries (other than missing material) should be directed to the *New Phytologist* Central Office.

## Data Availability

All data used in this study are available in the Dataset S1.
