# Supplementary material for: Intra‐annual transfer of hydrogen and oxygen isotopic signals from water and sugar precursors to tree rings: Processes and mechanisms
Source: New Phytol. 2026 May 13;251(3):1143–60. doi: 10.1111/nph.71260 (PMC13326508; doi:10.1111/nph.71260)
Supplement: Supplementary file 2 — Fig. S1 Simplified schematic of major reactions associated with isotope effects during heterotrophic metabolism in the sink‐cell cytosol. Fig. S2 Seasonal variations in δ18O and δ2H of phloem sugar, xylem sugar, and xylem starch in beech and spruce in 2021 and 2022. Fig. S3 Relationships between NSC concentration and isotope composition for xylem sugar and xylem starch in beech and spruce in 2021 and 2022. Fig. S4 Relationships between tree‐ring cellulose and phloem sugar, xylem sugar, and xylem starch for δ18O and δ2H isotope compositions in beech and spruce across 2021 and 2022. Fig. S5 Relationships between tree‐ring cellulose isotope composition and modelled leaf water or measured xylem water for δ18O and δ2H in beech and spruce during the 2021 and 2022 growth periods. Fig. S6 Relationships between the fraction of isotopic exchange and T ai, VPD, and RH in beech and spruce during the 2021 and 2022 growth periods. Fig. S7 Relationships between the fraction of isotopic exchange and VPD or xylem NSC concentration in beech and spruce during the 2021 and 2022 growth periods. Fig. S8 Relationships between δ18O and δ2H of xylem water, leaf water, and tree‐ring cellulose in beech and spruce during the 2021 and 2022 growth periods. Notes S1 Sensitivity analysis. Please note: Wiley is not responsible for the content or functionality of any Supporting Information supplied by the authors. Any queries (other than missing material) should be directed to the New Phytologist Central Office. [file NPH-251-1143-s001.pdf]

***New Phytologist* Supporting Information**

Article title: Intra-annual transfer of hydrogen and oxygen isotopic signals from water and sugar precursors to tree rings: Processes and mechanisms
Authors: Haoyu Diao, Meisha Holloway-Phillips, Xin Song, Fabian Bernhard, Peter Waldner, Kerstin Treydte, Matthias Saurer, Georg von Arx, Arthur Gessler, Katrin Meusburger, Marco M. Lehmann
Article acceptance date: 29 April 2026

The following Supporting Information is available for this article:

Fig. S1 Simplified schematic of major reactions associated with isotope effects during heterotrophic metabolism in the sink-cell cytosol.
Fig. S2 Seasonal variations in $\delta^{18}O$ and $\delta^{2}H$ of phloem sugar, xylem sugar, and xylem starch in beech and spruce in 2021 and 2022.
Fig. S3 Relationships between NSC concentration and isotope composition for xylem sugar and xylem starch in beech and spruce in 2021 and 2022.
Fig. S4 Relationships between tree-ring cellulose and phloem sugar, xylem sugar, and xylem starch for $\delta^{18}O$ and $\delta^{2}H$ isotope compositions in beech and spruce across 2021 and 2022.
Fig. S5 Relationships between tree-ring cellulose isotope composition and modelled leaf water or measured xylem water for $\delta^{18}O$ and $\delta^{2}H$ in beech and spruce during the 2021 and 2022 growth periods.
Fig. S6 Relationships between the fraction of isotopic modification and T_{ai}, VPD, and RH in beech and spruce during the 2021 and 2022 growth periods.
Fig. S7 Relationships between the fraction of isotopic modification and VPD or xylem NSC concentration in beech and spruce during the 2021 and 2022 growth periods.
Fig. S8 Relationships between $\delta^{18}O$ and $\delta^{2}H$ of xylem water, leaf water, and tree-ring cellulose in beech and spruce during the 2021 and 2022 growth periods.
Notes S1 Sensitivity analysis.

Fig. S1 Simplified schematic of major reactions associated with isotope effects during heterotrophic metabolism in the sink-cell cytosol. Blue cycles indicate compounds in which carbonyl-oxygen isotope exchange can occur during reactions. Enzymes shown in green catalyse isotopic exchange at carbon-bound hydrogen positions and facilitate oxygen isotopic exchange. Enzymes shown in pink are associated with hydrogen metabolism steps that involve kinetic isotope effects (KIEs). **Abbreviations:** dihydroxyacetone (DHAP); fructose 6-phosphate (F6P); fructose 1,6-bisphophate (FBP); glucose 6-phosphate (G6P); glucose-6-phosphate dehydrogenase (G6PDH); glyceraldehyde 3-phosphate (GAP); glyceraldhyde 3-phosphate dehydrogenase (GAPDH); hexokinase (HK); oxidative pentose phosphate pathway (OPPP); phosphoglucose isomerase (PGI); phosphoglucomutase (PGM); sucrose synthase (SuSy); triosephosphate isomerase (TPI); uridine diphosphate glucose (UDP-glucose).

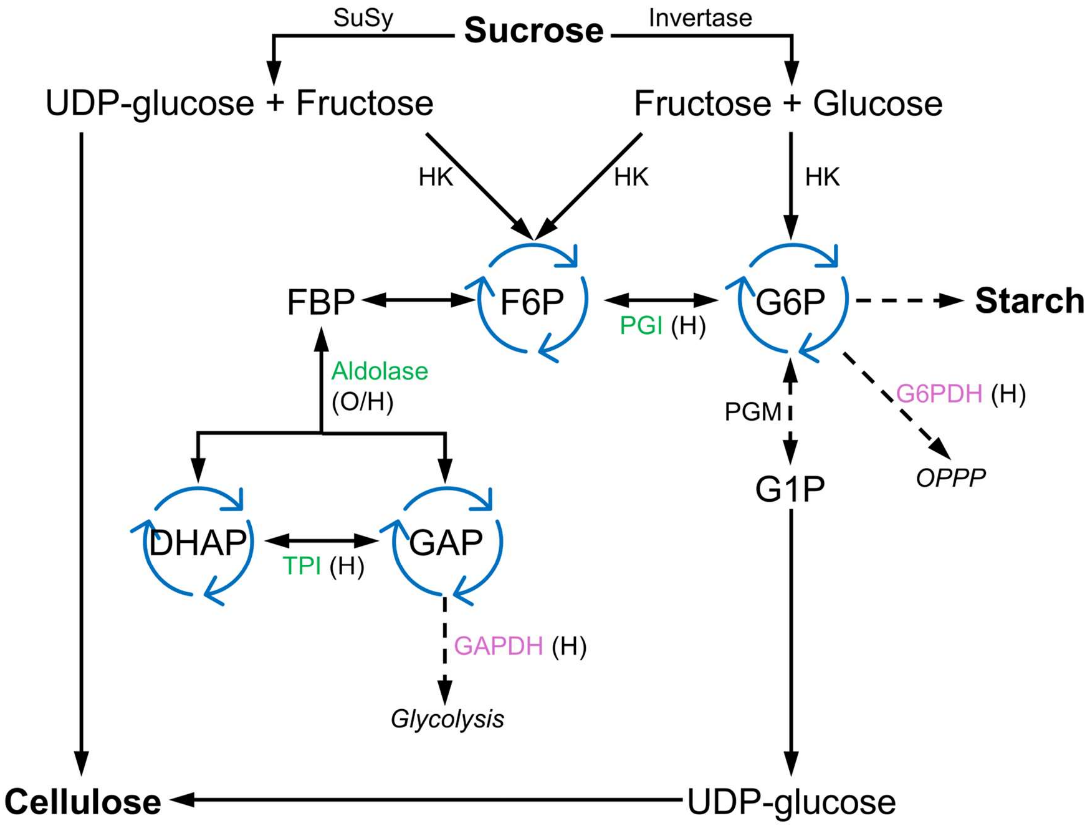

Fig. S2 Seasonal variations in oxygen ($\delta^{18}O$) and hydrogen ($\delta^{2}H$) isotope compositions of phloem sugar (δ_{PS}; a, b), xylem sugar (δ_{XS}; c, d), and xylem starch (δ_{XSt}; e, f) in beech and spruce in 2021 and 2022. Different colours indicate species; coloured lines represent smoothed trends. Growth periods are marked by vertical dashed lines.

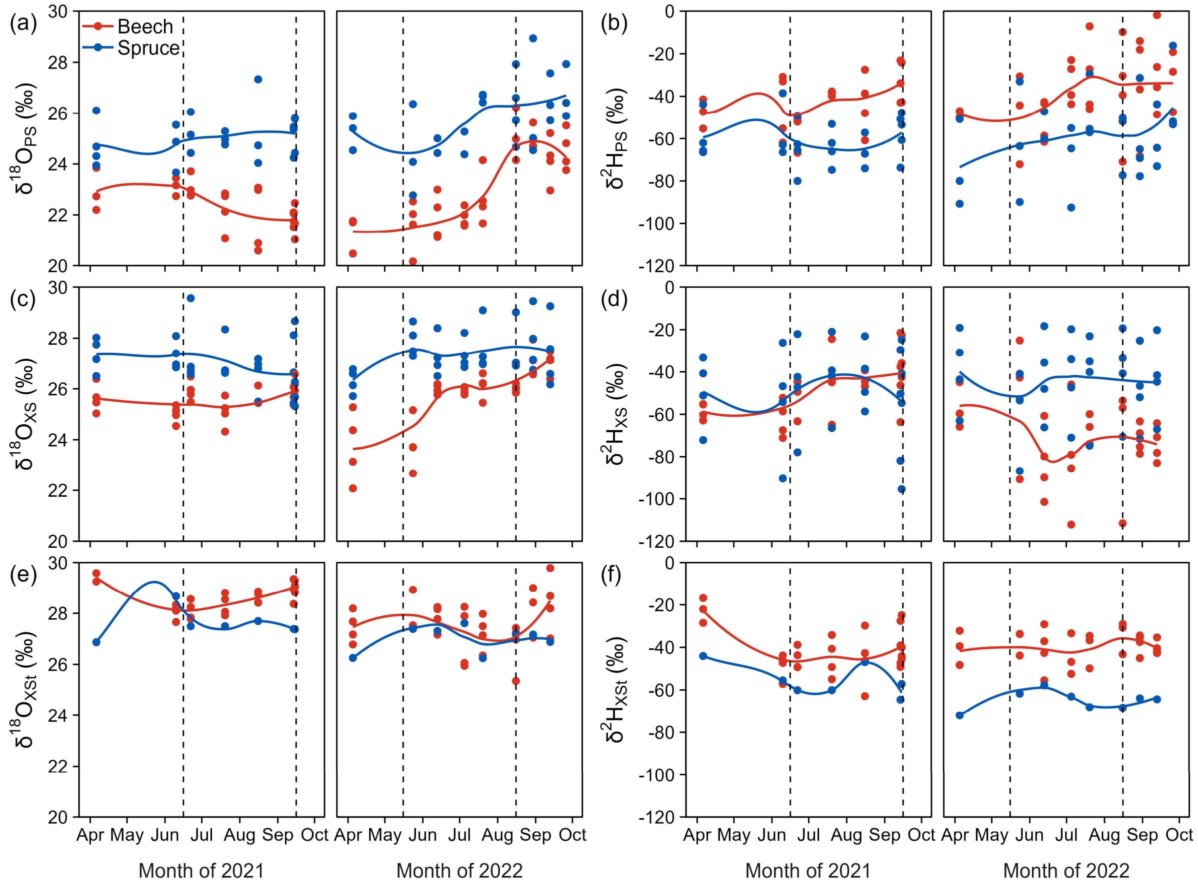

Fig. S3 Relationships between NSC concentration and isotope composition for xylem sugar and xylem starch in beech and spruce in 2021 and 2022. Panels show relationships between NSC concentration and oxygen ($\delta^{18}O$) or hydrogen ($\delta^{2}H$) isotope composition for xylem sugar (XS; a, b) and xylem starch (XSt; c, d), as well as relationships between xylem NSC concentration and apparent isotope fractionation between xylem sugar and xylem starch for oxygen (e; ε^{O}_{XS-XSt}) and hydrogen (f; ε^{H}_{XS-XSt}). Colours indicate species; symbols indicate years. Solid lines represent significant ($P < 0.05$) linear relationships fitted across years for each species. *P*-values and R^2 are shown for significant relationships only.

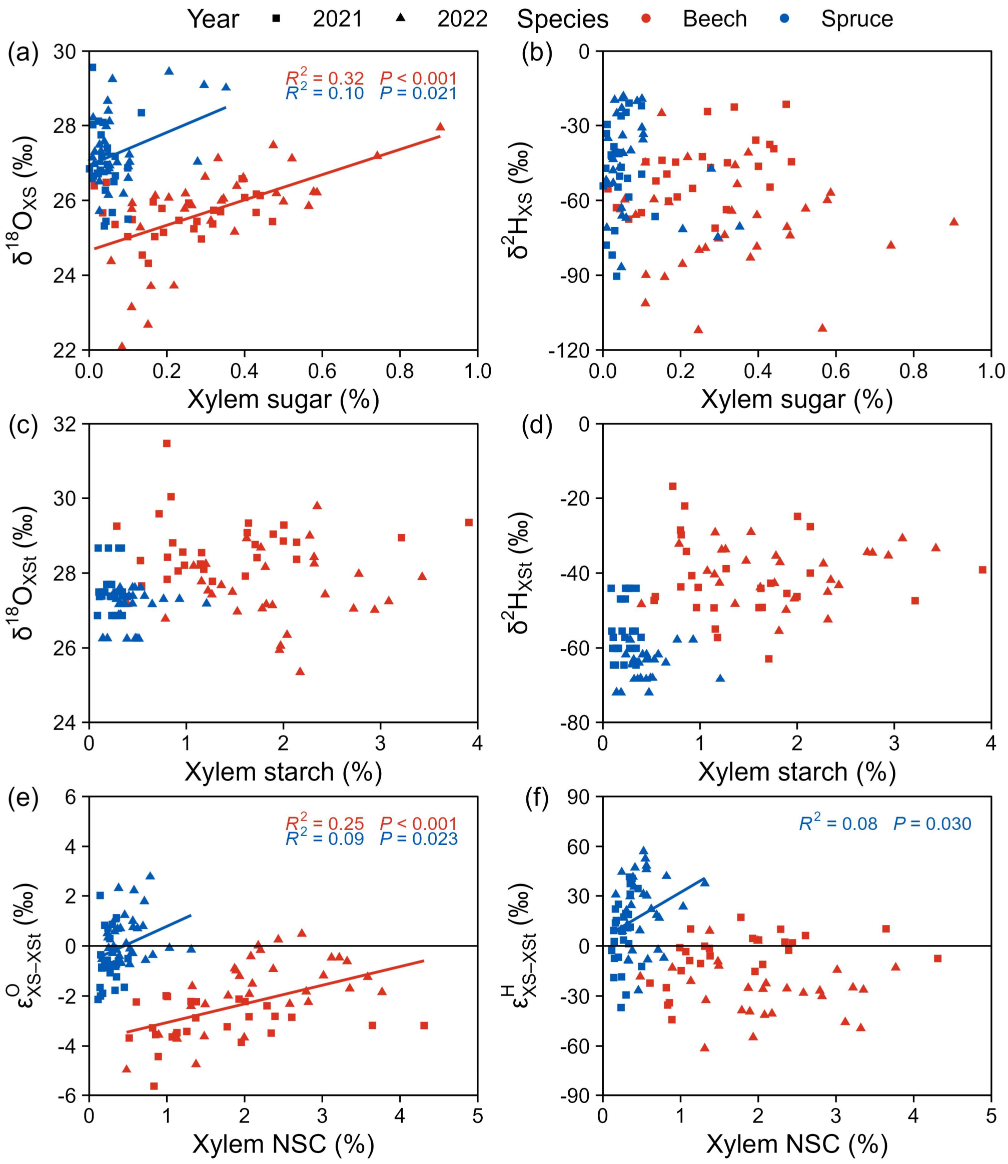

Fig. S4 Relationships between tree-ring cellulose and phloem sugar (PS), xylem sugar (XS), and xylem starch (XSt) for oxygen ($\delta^{18}O$; a–c) and hydrogen (δ^2H; d–f) isotope compositions in beech and spruce across 2021 and 2022. Data represent mean ± SD. Solid lines indicate significant (P < 0.05) linear relationships; dashed lines indicate non-significant linear relationships. Relationships are fitted across years for each species. Corresponding P-values and R^2 values are provided.

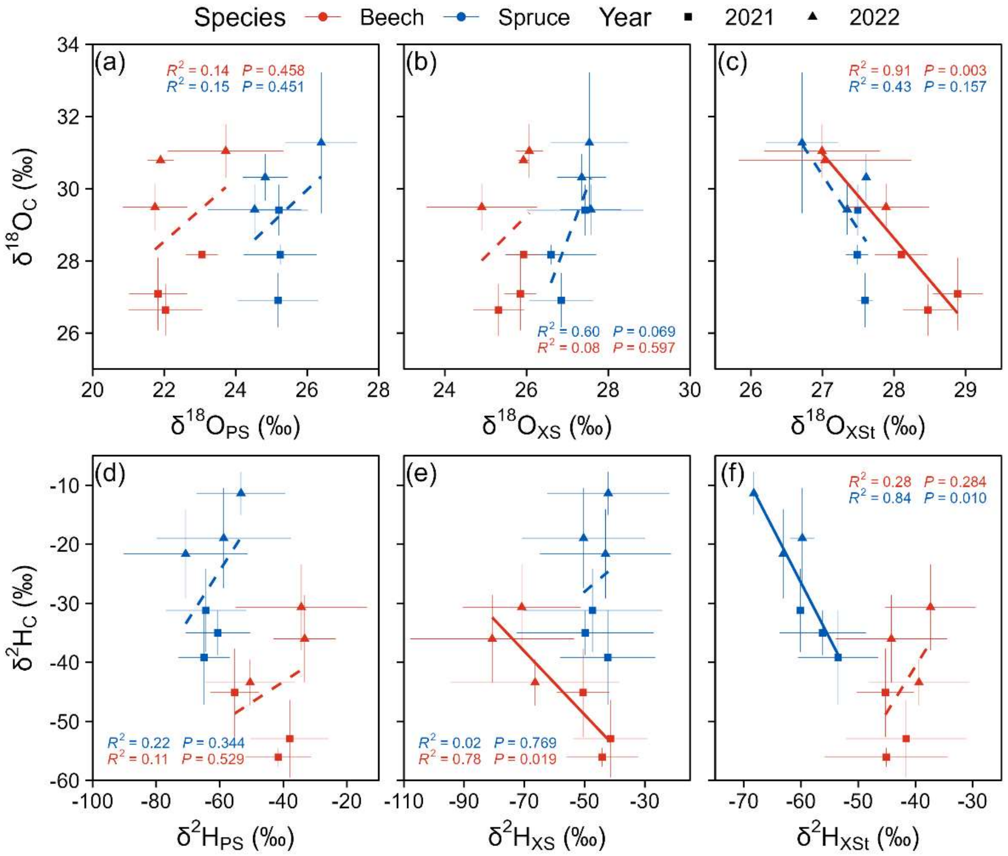

Fig. S5 Relationships between tree-ring cellulose isotope composition (δ_C) and modelled leaf water (δ_{LW}) or measured xylem water (δ_{XW}) for oxygen ($\delta^{18}O$) and hydrogen (δ^2H) in beech and spruce during the 2021 and 2022 growth periods. Different colours indicate species. Solid lines show significant linear relationships fitted within years for each species. *P*-values and R^2 values are provided.

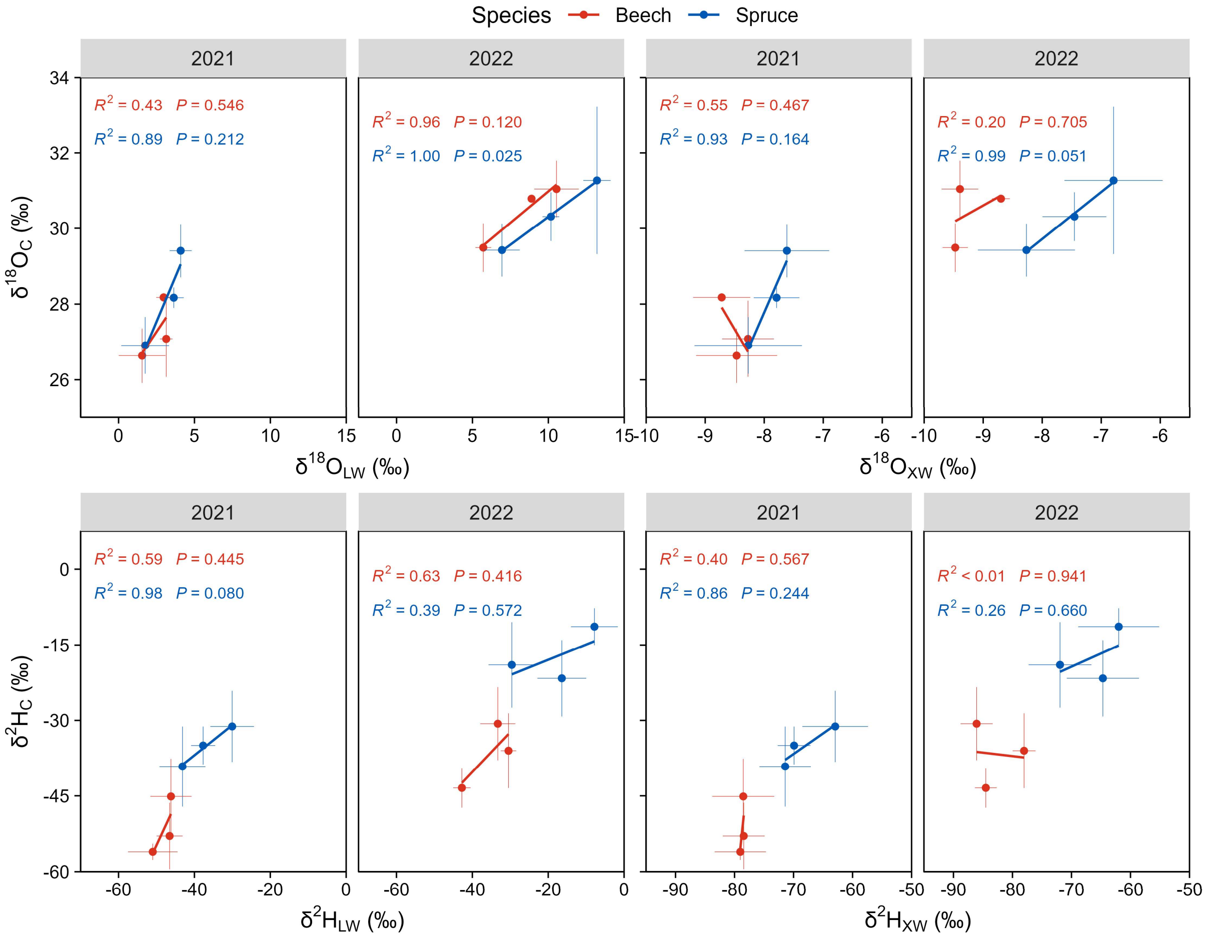

Fig. S6 Relationships between the fraction of isotopic modification (f) and air temperature (T_{air}; a, b), vapour pressure deficit (VPD; c, d), and relative humidity (RH; e, f) in beech and spruce during the 2021 and 2022 growth periods. Colours indicate species; symbols indicate years. Solid lines and grey bands show significant (P < 0.05) linear relationships and their 95% confidence intervals, fitted across years for each species. Regression equations, P-values, and R^2 values are provided for significant relationships only.

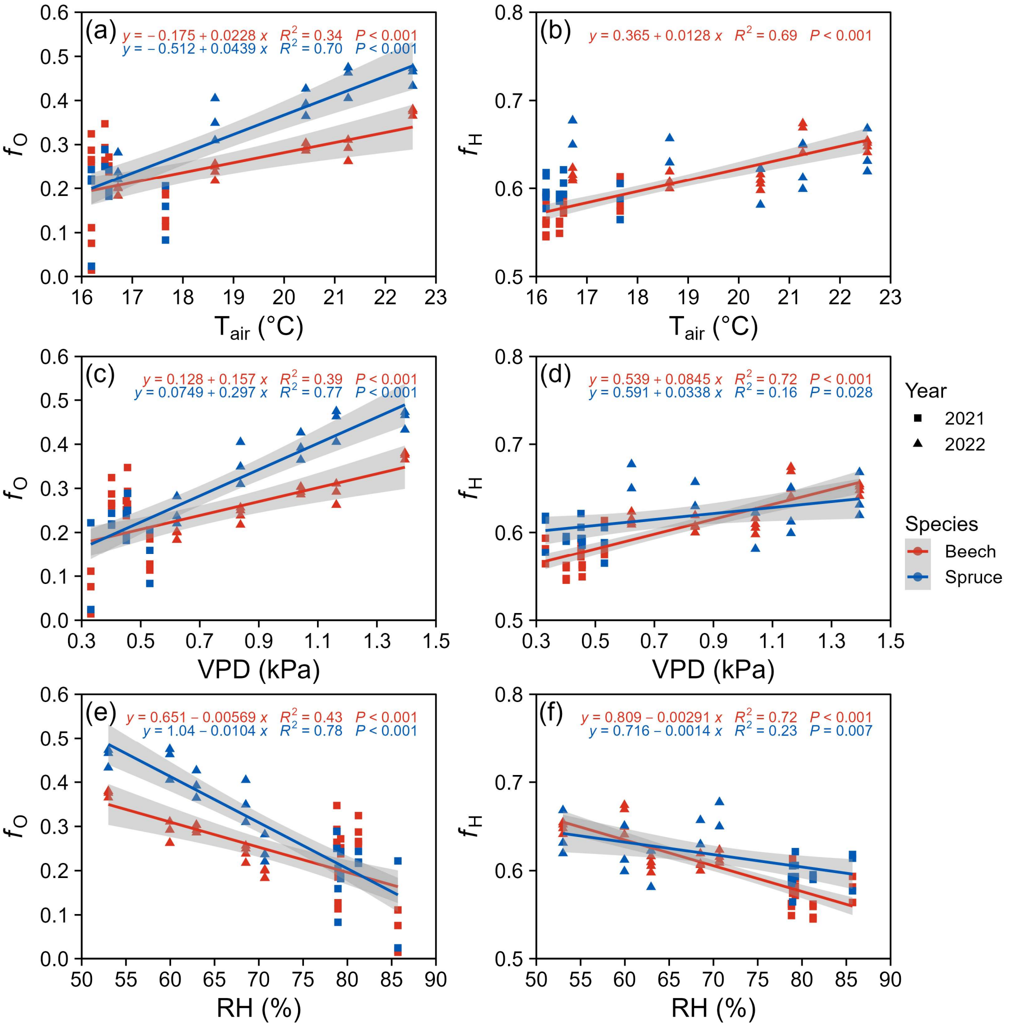

Fig. S7 Relationships between the fraction of isotopic modification (*f*) and vapour pressure deficit (VPD; a, b) or xylem NSC concentration (c, d) in beech and spruce during the 2021 and 2022 growth periods. Colours indicate species. Solid lines and grey bands represent significant ($P < 0.05$) linear relationships and their 95% confidence intervals, fitted separately by species and year. *P*-values and R^2 values are provided for significant relationships only.

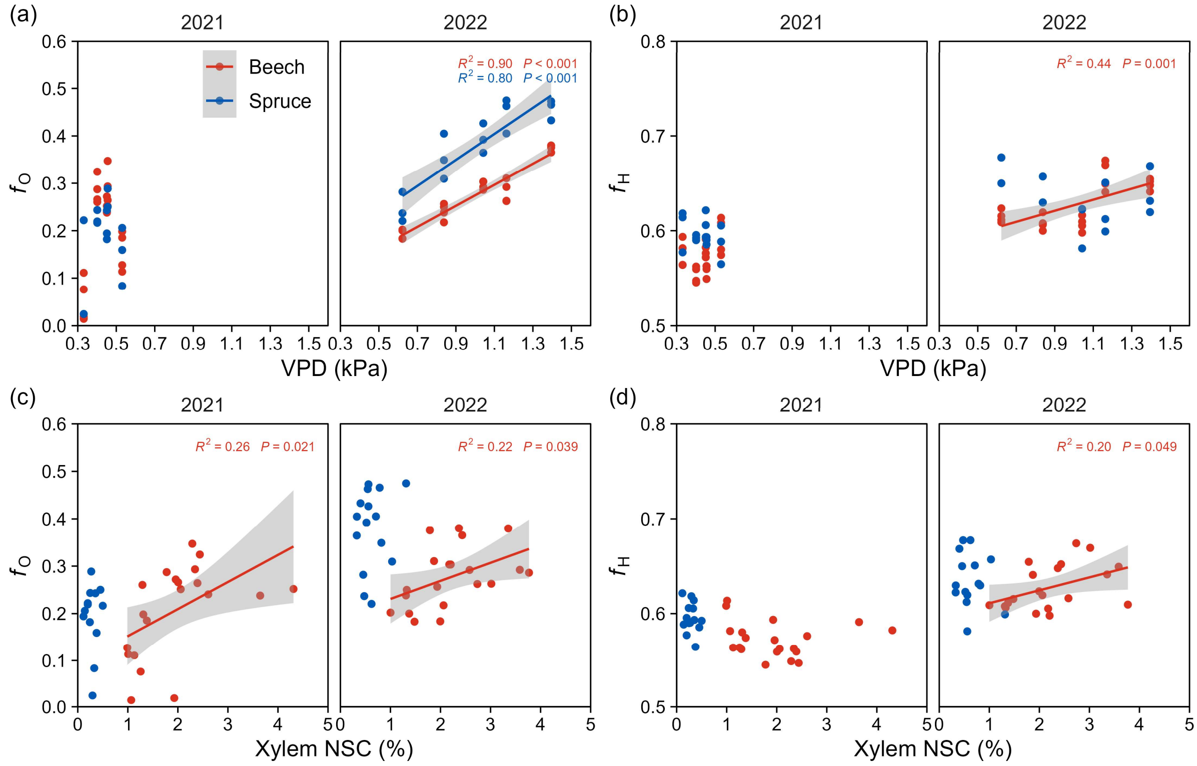

Fig. S8 Relationships between oxygen ($\delta^{18}O$) and hydrogen (δ^2H) isotope compositions of xylem water (δ_{XW}; a), leaf water (δ_{LW}; b), and tree-ring cellulose (δ_C; c) in beech and spruce during the 2021 and 2022 growth periods. Data represent mean ± SD. Different colours indicate species. Solid lines represent linear relationships fitted within years for each species. *P*-values and R^2 values are provided.

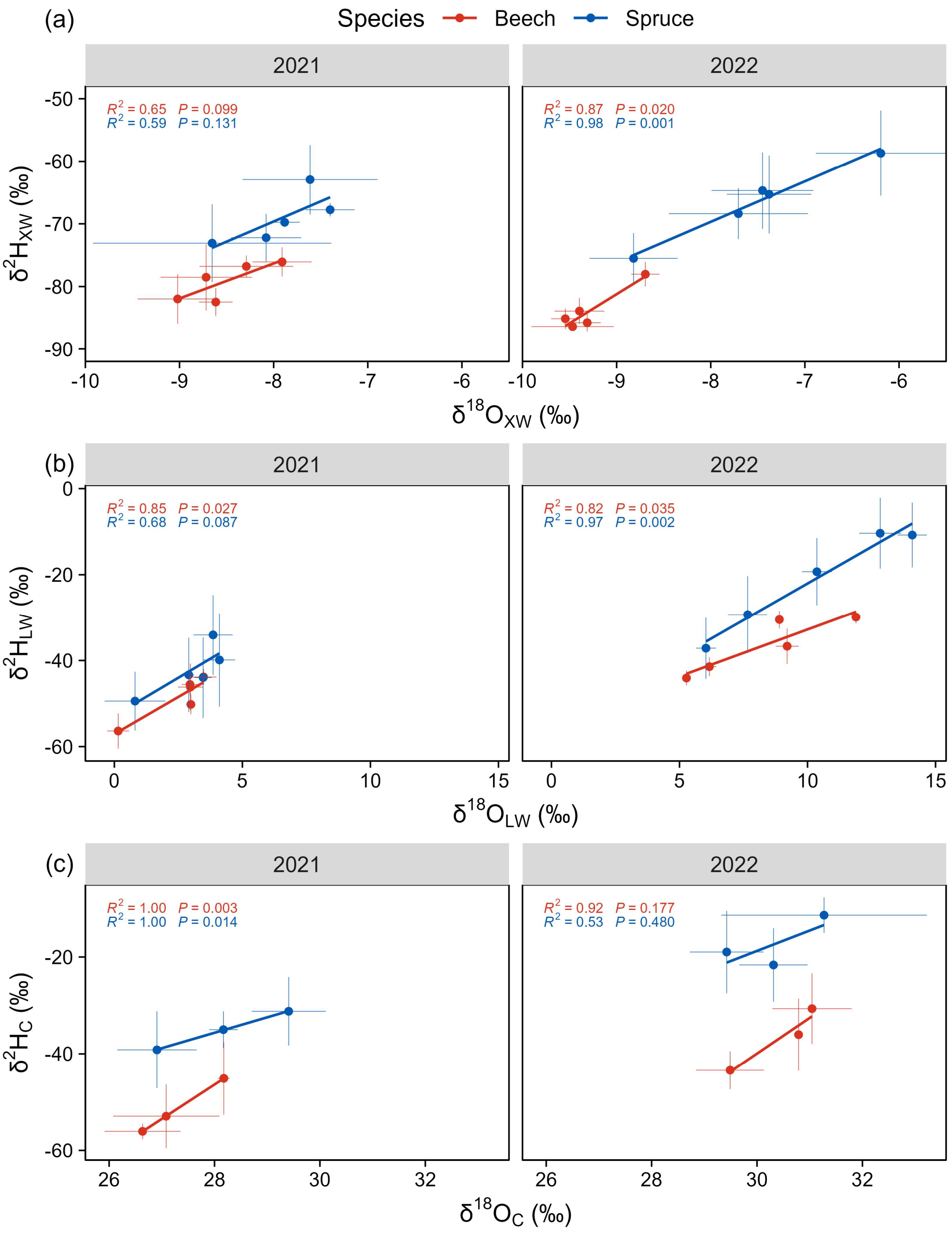

Notes S1 Sensitivity analysis

In calculating *f*, two parameters in Eqn 2 (ε_A and ε_H) were assumed to be constant during the two consecutive growth periods. To assess how this assumption influences the results, we performed a sensitivity analysis quantifying how variations in ε_A and ε_H affect the calculated *f* values.

Differentiation of Eqn 2 with respect to ε_A and ε_H yields:

$$\begin{cases} \dfrac{\partial f}{\partial \varepsilon_A} = \dfrac{(\delta_{LW} + 1000)[\delta_C - \delta_{XW}(1 + \varepsilon_H) - 1000\varepsilon_H]}{[\delta_{XW}(1 + \varepsilon_H) - \delta_{LW}(1 + \varepsilon_A) + 1000(\varepsilon_H - \varepsilon_A)]^2} \\ \dfrac{\partial f}{\partial \varepsilon_H} = -\dfrac{(\delta_{XW} + 1000)[\delta_C - \delta_{LW}(1 + \varepsilon_A) - 1000\varepsilon_A]}{[\delta_{XW}(1 + \varepsilon_H) - \delta_{LW}(1 + \varepsilon_A) + 1000(\varepsilon_H - \varepsilon_A)]^2} \end{cases} \quad \text{Eqn S1}$$

These equations apply to both oxygen and hydrogen. To calculate the sensitivities of f_O and f_H under realistic conditions, we used the parameter values in Table S1. The resulting values represent the absolute sensitivity of *f* to ε_A or ε_H, when ε_A or ε_H vary around their commonly accepted values. Because these same values were applied in *f* calculations in Eqn 2, this analysis quantifies uncertainties in Fig. 7 arising from possible variation in ε_A or ε_H. The resulting absolute sensitivities of *f* to ε_A or ε_H are shown in Table S2.

Changes in *f* values (Δf) due to changes in ε_A or ε_H ($\Delta\varepsilon_A$ or $\Delta\varepsilon_H$) were calculated as:

$$\Delta f = \frac{\partial f}{\partial \varepsilon_A \text{ or } \partial \varepsilon_H}(\Delta\varepsilon_A \text{ or } \Delta\varepsilon_H) \quad \text{Eqn S2}$$

The results (Table S3) show that the largest effect arises from $\Delta\varepsilon_A$ on Δf_O; however, a 1% change in ε_A results in only a 0.0133 change in f_O. Variations in ε_A and ε_H produce smaller changes in f_H than in f_O. A 50% change in ε_A yields a Δf_O of 0.6653 but only 0.1196 for Δf_H, while a 50% change in ε_H leads to Δf_O and Δf_H values below 0.3. We therefore conclude that *f* estimates based on Eqn 2 are relatively insensitive to plausible variations in ε_A and ε_H.

Table S1 Parameter values used in Eqn S1. δ_{XW}, δ_{LW}, and δ_C represent the oxygen or hydrogen isotope compositions of xylem water, leaf water, and tree-ring cellulose, respectively. Values are averaged across both species (beech and spruce) and both growth periods (2021 and 2022) at the study site (i.e., derived from Fig. 5). ε_A and ε_H are the autotrophic and heterotrophic isotope fractionation factors (unitless). The ε_A and ε_H values for oxygen and hydrogen are taken

from pioneering studies (Sternberg & DeNiro, 1983; Yakir & DeNiro, 1990) and are widely used in subsequent research.

	δ_{XW} (‰)	δ_{LW} (‰)	δ_{C} (‰)	ε_A	ε_H
Oxygen	–8.39	5.91	28.95	0.027	0.027
Hydrogen	–75.65	–36.34	–36.60	–0.171	0.158

Table S2 Absolute sensitivity of the isotopic modification fractions (*f* values) to autotrophic and heterotrophic isotope fractionation factors (ε_A and ε_H) calculated for both oxygen and hydrogen isotopes (unitless). The differentiations were calculated using values shown in Table S1.

$\frac{\partial f_O}{\partial \varepsilon_A}$	$\frac{\partial f_O}{\partial \varepsilon_H}$	$\frac{\partial f_H}{\partial \varepsilon_A}$	$\frac{\partial f_H}{\partial \varepsilon_H}$
49.28	18.94	–1.40	–2.06

Table S3 Changes in the isotopic modification fractions values (Δf) resulting from 1% and 50% variations in autotrophic or heterotrophic isotope fractionation factors ($\Delta\varepsilon_A$ or $\Delta\varepsilon_H$) for both oxygen and hydrogen.

	$\lvert\Delta\varepsilon_A\rvert$ = 1% ε_A	$\lvert\Delta\varepsilon_H\rvert$ = 1% ε_H	$\lvert\Delta\varepsilon_A\rvert$ = 50% ε_A	$\lvert\Delta\varepsilon_H\rvert$ = 50% ε_H
$\lvert\Delta f_O\rvert$	0.0133	0.0051	0.6653	0.2557
$\lvert\Delta f_H\rvert$	0.0024	0.0034	0.1196	0.1630
